# Difluoro-and Trifluoromethylation of Electron-Deficient Alkenes in an Electrochemical Microreactor

**DOI:** 10.1002/open.201300039

**Published:** 2013-11-26

**Authors:** Kenta Arai, Kevin Watts, Thomas Wirth

**Affiliations:** [a] School of Chemistry, Cardiff University,Park Place, Main Building, Cardiff CF10 3AT (United Kingdom)http://www.cf.ac.uk/chemy/wirth

**Keywords:** difluoroacetic acid, electrochemistry, flow chemistry, microreactor, radicals, trifluoroacetic acid

## Abstract

Electrochemical microreactors, which have electrodes integrated into the flow path, can afford rapid and efficient electrochemical reactions without redox reagents due to the intrinsic properties of short diffusion distances. Taking advantage of electrochemical microreactors, Kolbe electrolysis of di-and trifluoroacetic acid in the presence of various electron-deficient alkenes was performed under constant current at continuous flow at room temperature. As a result, di-and trifluoromethylated compounds were effectively produced in either equal or higher yields than identical reactions under batch conditions previously reported by Uneyamas group. The strategy of using electrochemical microreactor technology is useful for an effective fluoromethylation of alkenes based on Kolbe electrolysis in significantly shortened reaction times.

## Introduction

Difluoromethyl (CHF_2_) and trifluoromethyl (CF_3_) groups are important structural motifs in many pharmaceutically relevant molecules because they are known to enhance chemical and metabolic stability, lipophilicity and binding selectivity.[1a] As a result, much effort has been directed toward the development of facile and efficient synthetic methods for the introduction of CHF_2_ and CF_3_ groups into aromatics and heteroaromatics as well as alkenes. Increased effort has been focused on the development of new reagents, which can be effective sources of reactive radicals or nucleophilic species of CHF_2_ and CF_3_, such as Tognis,[2] Umemotos,[3] and Barans reagent[4] and application of those reagents to copper-catalyzed trifluoromethylation of unactivated alkenes to enable the formation of a sp^3^-C−CF_3_ bond.[5]

Uneyama and co-workers previously reported the electrooxidation of trifluoroacetic acid (TFA) in the presence of various alkenes for the preparation of trifluoromethylated aliphatic compounds.[6] TFA is of low cost and the generated CF_3_ radicals based on Kolbe electrolysis can efficiently react with various electron-deficient alkenes in a batch system.[6] Very recently, Buchwald and Chen reported a rapid and efficient protocol for trifluoromethylation of aryl and heteroaryl iodides using potassium trifluoroacetate as a source of CF_3_ radicals in a continuous flow system, which enabled a rapid rate of reaction.[7] On the basis of these results it is proposed that an electrochemical trifluoromethylation might be improved by using a flow system.

**Figure 1:**
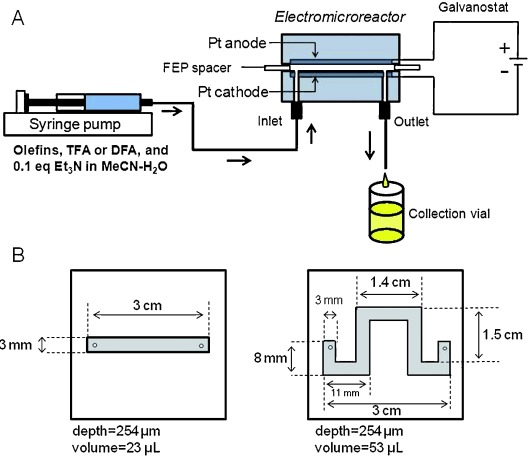
A) Flow setup for electrochemical di-and trifluoromethylation. B) FEP micro flow channel.

We recently reported a practical procedure for electrochemical syntheses including dimerization of diphenylacetic acid coupled with Kolbe electrolysis by using an electrochemical microreactor, combining the advantages of flow and electrochemistry.[8] The electrochemical microreactor has a flow channel sandwiched between two platinum electrodes as shown in Figure 1, where very short diffusion distances lead to high space-time yields (see Supporting Information for more details on the electrochemical microreactor).

In short, it enables a rapid and effective progress of oxidative and reductive reactions without redox reagent because the anode and cathode act as oxidant and reductant, respectively, during the passage of reactants through the flow channel. Different microreactor systems have already been developed for chemistry and have been successfully used for electrochemical reactions without added electrolyte.[9] In verity, some synthetic protocols and other examples for practical use of such reactors have been reported.[10]

## Results and Discussion

### Kolbe electrolysis

**Scheme 1:**
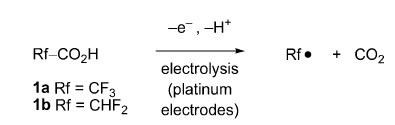
Anodic Kolbe electrolysis of di-and trifluoroacetic acid.

In this paper, we report the rapid and efficient electrochemical addition of CHF_2_ and CF_3_ radicals to various electron-deficient alkenes. The radicals are produced by Kolbe electrolysis of di-and trifluoroacetic acid, respectively, at the platinum anode (see Scheme 1). The Kolbe electrolysis of trifluoroacetic acid in the presence of electron-deficient alkenes such as acrylates and acryl amides in batch conditions has been reported in the literature.[6], [11] Herein we describe the usefulness of the flow protocols for di-and trifluoromethylation on the basis of these earlier results.

**Scheme 2:**
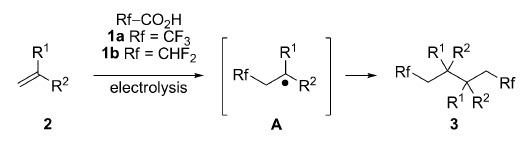
Di-and trifluoromethylation of olefins.

A typical electrochemical di-and trifluoromethylation of alkenes **2** in acetonitrile/water as a solvent mixture is shown in Scheme 2. The addition of radical **1 a** or **1 b** to alkenes **2** is followed by dimerization of the radical intermediates **A** to yield products **3**.[6], [12]

### Optimization

To demonstrate the usability of the electrochemical microreactor for radical trifluoromethylation, we initially examined the dimerization by using methyl acrylate **2 a** (R^1^=CO_2_Me, R^2^=H) as the substrate, which is a well-established reaction in batch chemistry.[6] The electrolysis was performed by continuous introduction of a water/acetonitrile solution containing **2 a**, trifluoroacetic acid and triethylamine into the electrochemical microreactor with a flow channel (23 μL volume; Figure 1, left) at constant current and room temperature (Figure 1). The reaction conditions were optimized by preliminary experiments. Hydrogen is formed at the cathode through proton reduction and is visible at the exit of the reactor; however, the amounts generated do not disturb the reaction. After purification, dimer **3 a** was obtained in 52 % yield as a 5:3 mixture of dl and *meso* isomers as determined by ^13^C NMR spectroscopy. This yield is comparable to previous reports,[6] although the reaction time with 66 s was much shorter than in the batch reaction (16 h). Similarly, the electrolysis of trifluoroacetic acid in the presence of ethyl acrylate **2 b** (R^1^=CO_2_Et, R^2^=H), *tert*-butyl acrylate **2 c** (R^1^=CO_2_*t*Bu, R^2^=H), or methyl methacrylate **2 d** (R^1^=CO_2_Me, R^2^=Me) afforded dimers **3 c**, **3 e** and **3 g** as a mixture of dl and *meso* isomers in the ratio of 1:1, 1:1 and 10:1, in 45, 45 and 11 % yield, respectively. It has been previously reported by Renaud et al. that dimer **3 g** can be obtained in about 10 % isolated yield by electrolysis under batch conditions.[11a] Under batch conditions, the dimers are typically formed in a 1:1 dl:*meso* ratio.

In order to expand the feasibility of the Kolbe electrolysis using other acids (**1**) as a source of fluoromethyl radicals, the reaction conditions optimized for the trifluoromethylation of **2 a** were applied to the electrolysis using difluoroacetic acid **1 b** in the presence of acrylates **2**. As a result, similar difluoromethylated dimers **3 b**, **3 d**, **3 f** and **3 h** were obtained as summarized in Table 1.

**Table 1:** Di-and trifluoromethylation of acrylates.

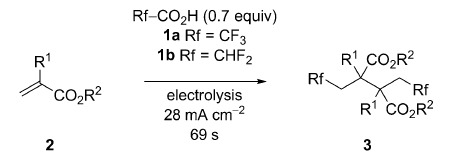					
Entry	Acid 1	Alkene 2	Product	dl:*meso*	Yield [%]
1	**1 a**	**2 a** R^1^=H, R^2^=Me	**3 a**	5:3	52[a]
2	**1 b**	**2 a** R^1^=H, R^2^=Me	**3 b**	1:1	38[b]
3	**1 a**	**2 b** R^1^=H, R^2^=Et	**3 c**	1:1	45
4	**1 b**	**2 b** R^1^=H, R^2^=Et	**3 d**	1:1	40
5	**1 a**	**2 c** R^1^=H, R^2^=*t*Bu	**3 e**	1:1	45
6	**1 b**	**2 c** R^1^=H, R^2^=*t*Bu	**3 f**	1:1	40
7	**1 a**	**2 d** R^1^=Me, R^2^=Me	**3 g**	10:1[c]	11[d]
8	**1 b**	**2 d** R^1^=Me, R^2^=Me	**3 h**	6:5[e]	16

[a] Batch yield: 50 % (1:1 dl:*meso*), ref. [6].

[b] Batch yield: 45 % (1:1 dl:*meso*), ref. [11e].

[c] or 1:10.

[d] Batch yield: 10 % (1:1 dl:*meso* ), ref. [11a].

[e] or 5:6.

### Di-and trifluoromethyl acetamidation

**Scheme 3:**
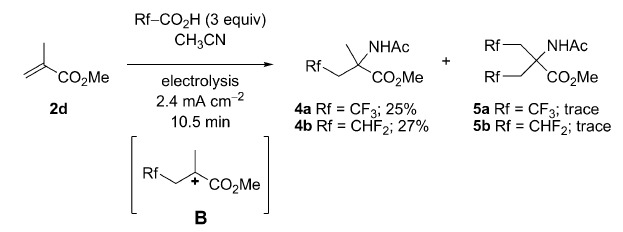
Di-and trifluoromethylation of methyl methacrylate 2 d.

Changing the substituents on the C=C double bond of alkenes should affect not only the yield but also the selectivity in the product due to the modification of the thermodynamic and kinetic stability of the radical intermediates **A** and their affinity to the electrodes.[6] When the electrolysis of trifluoroacetic acid **1 a** in the presence of **2 d** was carried out under the above-described conditions for the dimerization, not only **3 g**, but also small amounts of **4 a** and **5 a**, obtained through a 1,2-addition of trifluoromethyl and acetamido groups, were observed by ^1^H NMR analysis as shown in Scheme 3. It has been reported that this type of reaction can occur from nucleophilic attack of acetonitrile to the carbocation **B** during the electrolysis of trifluoroacetic acid in the presence of **2 d** under low current densities (1 mA cm^−2^) at 0 °C.[11b]

To investigate the trifluoromethyl acetamidation in flow chemistry we examined other reaction conditions. Initially, similar reaction conditions as described in the literature were applied in the flow reaction at a flow rate of 0.05 mL min^−1^ (residence time: 28 s). However, no conversion of **2 d** to **4 a** at this low current density (1 mA cm^−2^) was observed. When the current was raised to 50 mA cm^−2^, the two amide-proton signals corresponding to **4 a** and **5 a** at 6.45 ppm and 6.68 ppm, respectively, were clearly observed by ^1^H NMR. This is consistent with the previous report by Uneyama et al.[11b] The products were observed in the crude reaction mixture in a ratio of 2:3 and 3:1 (**4 a**:**5 a**) at residence times of 22 s and 13 s, respectively. From these experiments, it can be concluded that the initially generated mono-trifluoromethyl-substituted product **4 a** can undergo conversion to **5 a** through a different radical intermediate. However, from the crude NMR spectra of the reactions, it is obvious that these products are only a minority within the crude reaction mixture.

To establish a suitable protocol for the trifluoromethyl acetamidation, which is a useful reaction for the synthesis of *α*-methyl amino acids as building blocks for peptide and protein synthesis,[13] we further investigated the trifluoromethylation of **2 d** using other reaction conditions. The electrolysis in the electrochemical microreactor using a larger flow channel (53 μL; Figure 1, right) at the flow rate of 5 μL min^−1^ (residence time: 10.5 min) and a low current density (2.4 mA cm^−2^) led without dimerization to the preferential generation of **4 a** and **4 b** in 25 % and 27 % isolated yields, respectively. The trifluoroacetamidation of **2 d** giving 20 % isolated yield of **4 a** under batch conditions was previously achieved in 8 h reaction time at 0–5 °C.[11b] In our experiments, the isolated yield was improved using much shorter reaction times at room temperature. Thus, the results of electrolysis by using **2 d** as a substrate clearly showed that the electrochemical microreactor is suitable not only for radical dimerization of acrylates, but also for nucleophilic addition of acetonitrile accompanied with di-and trifluoromethylation. It should be noticed that a 1,2-addition of di-and trifluoromethyl and acetamido groups to acrylates **2 a**–**c** was not observed even when applying the conditions for the synthesis of **4 a** and **4 b**.

### Bis(difluoromethylation) and bis(trifluoromethylation)

**Scheme 4:**
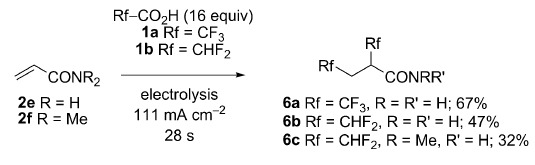
Di-and trifluoromethylation of acrylamide 2 e and 2 f.

In order to further demonstrate the usefulness of the electrochemical microreactor for di-and trifluoromethylation, the electrolysis of **1 a** and **1 b** in the presence of acrylamide **2 e** was investigated. According to the literature,[11c] the 1,2-addition of trifluoromethyl radicals as shown in Scheme 4 can be selectively progressed. The nitrogen atom of the radical intermediate **A** of acrylamide can strongly absorb on the platinum electrode surface, so that **A** diffuses slowly to the bulk solution and consequently reacts with another Rf radical on the electrode surface without dimerization, preferentially promoting bis(trifluoromethylation) when the current density is kept high.

It was previously demonstrated that bis(trifluoromethylation) to **6** can be achieved in 35 % yield without dimerization and trifluoroacetamidation by electrolysis of **2 e** in an undivided cell for 3.5 h at 200 mA cm^−2^ at 0 °C.[11c] In addition, Uneyama et al. reported that this reaction is successfully progressed in flow systems that employed a large flow cell with platinum electrodes to give 48 % yield of **6 a** (Rf=CF_3_, R=R′=H).[13] Thus, the examination of electrochemical bis(fluoromethylation) in a micro flow channel and comparison of the result with the previous report is important in evaluating the usability of our method. The best conditions for this reaction (see Experimental Section for details) were found to be the electrolysis of **2 e** with **1 a** and triethylamine in 7:1 acetonitrile/water at constant current (110 mA cm^−2^) at a flow rate of 0.05 mL min^−1^ (residence time=28 s) at room temperature. Compounds **6 a** and **6 b** (Rf=CHF_2_, R=R′=H) were selectively obtained in 67 % and 47 % yield, respectively, dramatically improving the reported yields.[11c], [13] Thus, these results further and strongly support that the electrochemical microreactor is a useful device to achieve rapid and effective di-and trifluoromethylation for electro-deficient alkenes based on Kolbe electrolysis.

The reaction conditions were also applied to the electrolysis of **1 a** and **1 b** in the presence of *N*,*N*-dimethylacrylamide **2 f**. In contrast to the high selectivity and yields of **6 a** and **6 b**, some other side products, which could not be identified by NMR analysis, were observed in this reaction. Only in the case of electrolysis of **1 b**, was the bis(difluoromethylation) accompanied by a reductive demethylation to yield **6 c** (Rf=CHF_2_, R=Me, R′=H) in 32 %.

## Conclusions

Taking advantage of an electrochemical microreactor, di-and trifluoromethylations of electron-deficient alkenes based on Kolbe electrolysis of di-and trifluoroacetic acid were performed in good yields, which either equal or surpass the yields obtained from experiments under batch conditions. We conclude that the protocols shown in this paper are useful for rapid and effective preparation of di-or trifluoromethylated compounds, as the reactions proceed in very short reaction times and at room temperature.

## Experimental Section

**General**: Melting points were obtained in open capillary tubes and are uncorrected. ^1^H NMR and ^13^C NMR spectra were recorded on an AV-400 Bruker spectrometer in the indicated solvents at 400 MHz. MS (EI) and HRMS were recorded using: EI: Finnigan MAT 95×P; APCI: Thermofisher LTQ Orbitrap XL; ESI: Waters 2Q4000. All reactions were monitored by thin-layer chromatography (TLC), which was performed on precoated sheets of silica gel 60 (Merck). The electrochemical microreactor, having Pt foil (0.1 mm) as anode and cathode and fluorinated ethylene polymer (FEP) channel as shown in Figure 1 was used. The galvanostatic reactions were performed with a GWINSTEC GPR-30H100. All other chemicals were used as purchased without further purification.

**General procedure for electrochemical dimerization**: A mixture of **1 a** or **1 b** (5.0 mmol), **2** (7.6 mmol), and Et_3_N (0.5 mmol, 51 mg) was dissolved in CH_3_CN/H_2_O (7:1 *v*/*v*; 5 mL) and introduced into the electrochemical microreactor equipped with a FEP channel (Figure 1, left) through a syringe pump (20 μL min^−1^; residence time: 69 s) with an applied current of 50 mA (current density: 28 mA cm^−2^) and collected in a glass vial at the outlet. After the solvents were removed by evaporation under reduced pressure, H_2_O (10 mL) was added to the residual yellow oil. The aqueous solution was extracted with EtOAc (3×10 mL). The resulting organic layer was washed with saturated aq NaHCO_3_ (25 mL) and brine (25 mL), dried over MgSO_4_, and concentrated in vacuo. The residual yellow oil was purified by column chromatograph (silica gel) equilibrated by a mixture of hexane and EtOAc as eluent. The isolated yields of **3** were determined on the basis of **1 a** or **1 b**.

**Dimethyl 2,3-bis(2,2,2-trifluoroethyl)succinate (3 a)**:[6] Following purification of the crude product using column chromatography (2:1 hexane/EtOAc), **3 a** was obtained as a colorless solid (collection of 5 mL; yield: 0.401 g, 52 %): mp (dl): 49–52.5 °C, lit.[11d] 53–54 °C; mp (*meso*): 86–89 °C, lit.[11d] 87–89 °C; ^1^H NMR (400 MHz, CDCl_3_): *δ*=3.70 (s, 6 H, dl), 3.69 (s, 6 H, *meso*), 3.04–2.96 (m, 2 H, dl and *meso*), 2.82–2.61 (m, 2 H, dl and *meso*), 2.41–2.23 (m, 2 H, dl), 2.17–2.05 (m, 2 H, *meso*) ppm; ^13^C NMR (100 MHz, CDCl_3_): *δ*=33.22 (q, *J*=29.4 Hz, CH_2_, dl), 33.62 (q, *J*=29.6 Hz, CH_2_, *meso*), 40.71 (q, *J*=3.5 Hz, CH, dl), 41.30 (q, *J*=2.6 Hz, CH, *meso*), 53.01 (s, CH_3,_ dl), 53.03 (s, CH_3_, *meso*), 125.87 (q, *J*_CF_=275.0 Hz, CF_3_, *meso*), 126.18 (q, *J*_CF_=274.9 Hz, CF_3_, dl), 171.36 (s, C=O, dl), 171.46 (s, C=O, *meso*) ppm; MS (APCI): *m*/*z* (%): 311.07 (100) [*M*+H]^+^.

**Dimethyl 2,3-bis(2,2-difluoroethyl)succinate (3 b)**:[10e] Following purification of the crude product using column chromatography (2:1 hexane/EtOAc), **3 b** was obtained as a colorless liquid (collection of 5 mL; yield: 0.262 g, 38 %): ^1^H NMR (400 MHz, CDCl_3_): *δ*=5.86 (tm, *J*_HF_=56.6 Hz, 2 H, dl and *meso*), 3.67 (s, 6 H, dl or *meso*), 3.66 (s, 6 H, dl or *meso*), 3.02–2.93 (m, 2 H, dl and *meso*), 2.39–2.15 (m, 2 H, dl and *meso*), 1.97–1.73 (m, 2 H, dl and *meso*) ppm; ^13^C NMR (100 MHz, CDCl_3_): *δ*=33.42 (t, *J*=22.2 Hz, CH, dl or *meso*), 33.76 (t, *J*=22.2 Hz, CH, dl or *meso*), 40.96 (dd, *J*=6.8, 10.9 Hz, CH_2_, dl or *meso*), 41.23 (dd, *J*=6.6, 10.7 Hz, CH_2_, dl or *meso*), 52.85 (s, CH_3_, dl or *meso*), 52.92 (s, CH_3_, dl or *meso*), 116.01 (t, *J*_CF_=237.9 Hz, CHF_2_, dl or *meso*), 116.11 (t, *J*_CF_=237.8 Hz, CHF_2_, dl or *meso*), 172.60 (s, C=O, dl or *meso*), 172.68 (s, C=O, dl or *meso*) ppm; MS (APCI): *m*/*z*: 275.09 (100) [*M*+H]^+^.

**Diethyl 2,3-bis(2,2,2-trifluoroethyl)succinate (3 c)**: Following purification of the crude product using column chromatography (3:1 hexane/EtOAc), **3 c** was obtained as a colorless liquid (collection of 5 mL; yield: 0.378 g, 45 %): ^1^H NMR (400 MHz, CDCl_3_): *δ*=4.18–4.10 (m, 4 H, dl and *meso*), 3.01–2.93 (m, 2 H, dl and *meso*), 2.81–2.61 (m, 2 H, dl and *meso*), 2.39–2.26 (m, 2 H, dl or *meso*), 2.17–2.05 (m, 2 H, dl or *meso*), 1.21 (t, *J*=7.3 Hz, 6 H, dl or *meso*) 1.20 (t, *J*=7.2 Hz, 6 H, dl or *meso*) ppm; ^13^C NMR (100 MHz, CDCl_3_): *δ*=14.28 (s, CH_3_, dl or *meso*), 14.31 (s, CH_3_, dl or *meso*), 33.27 (q, *J*=29.6 Hz, *C*H_2_CF_3_, dl or *meso*), 33.77 (q, *J*=29.6 Hz, *C*H_2_CF_3_, dl or *meso*), 41.13 (q, *J*=2.5 Hz, CH, dl or *meso*), 41.70 (q, *J*=2.5 Hz, CH, dl or *meso*), 62.36 (s, *C*H_2_CH_3_, dl or *meso*), 62.42 (s, *C*H_2_CH_3_, dl or *meso*), 126.29 (q, *J*_CF_=275.0 Hz, CF_3_, dl or *meso*), 126.18 (q, *J*_CF_=274.8 Hz, CF_3_, dl or *meso*), 172.13 (s, C=O, dl or *meso*), 171.22 (s, C=O, dl or *meso*) ppm; HRMS (EI) *m*/*z*: [*M*+H]^+^ calcd for C_12_H_17_F_6_O_4_: 339.1026, found: 339.1029.

**Diethyl 2,3-bis(2,2-difluoroethyl)succinate (3 d)**: Following purification of the crude product using column chromatography (3:1 hexane/EtOAc), **3 d** was obtained as a colorless liquid (collection of 5 mL; yield: 0.300 g, 40 %): ^1^H NMR (400 MHz, CDCl_3_): *δ*=5.96 (tm, *J*_HF_=55.4 Hz, 2 H, dl and *meso*), 4.27–4.14 (m, 4 H, dl and *meso*), 3.09–2.98 (m, 2 H, dl and *meso*), 2.47–2.27 (m, 2 H, dl and *meso*), 2.02–1.85 (m, 2 H, dl and *meso*), 1.32–1.24 (m, 6 H, dl and *meso*) ppm; ^13^C NMR (100 MHz, CDCl_3_): *δ*=14.25 (s, CH_3_, dl and *meso*), 33.12 (t, *J*=22.3 Hz, CH_2_, dl or *meso*), 33.59 (t, *J*=22.3 Hz, CH_2_, dl or *meso*), 40.83 (dd, *J*=7.0, 3.8 Hz, CH, dl or *meso*), 41.15 (q, *J*=6.5, 3.8 Hz, CH, dl or *meso*), 61.75 (s, CH_2_CH_3_, dl or *meso*), 61.86 (s, CH_2_CH_3_, dl or *meso*), 115.85 (t, *J*_CF_=238.0 Hz, CHF_2_, dl or *meso*), 115.96 (t, *J*_CF_=237.5 Hz, CHF_2_, dl or *meso*), 171.90 (s, C=O, dl or *meso*), 171.99 (s, C=O, dl or *meso*) ppm; HRMS (EI): *m*/*z*: [*M*+H]^+^ calcd for C_12_H_19_F_4_O_4_: 303.1216, found: 303.1214.

**Di-*tert*-butyl 2,3-bis(2,2,2-trifluoroethyl)succinate (3 e)**: Following purification of the crude product using column chromatography (5:1 hexane/EtOAc), **3 e** was obtained as a colorless oil (collection of 5 mL; yield: 0.440 g, 45 %): ^1^H NMR (400 MHz, CDCl_3_): *δ*=2.87–2.72 (m, 2 H, dl and *meso*), 2.70–2.57 (m, 2 H, dl and *meso*), 2.30–2.18 (m, 2 H, dl or *meso*), 2.13–2.01 (m, 2 H, dl or *meso*), 1.40 (s, 18 H) 1.39 (s, 18 H) ppm; ^13^C NMR (100 MHz, CDCl_3_): *δ*=26.80 (s, C(*C*H_3_)_3_, dl or *meso*), 26.83 (s, C(*C*H_3_)_3_, dl or *meso*), 31.46 (q, *J*=29.1 Hz, CH_2_, dl or *meso*), 32.68 (q, *J*=29.4 Hz, CH_2_, dl or *meso*), 40.46 (q, *J*=2.6 Hz, CH, dl or *meso*), 41.37 (q, *J*=2.5 Hz, CH, dl or *meso*), 81.78 (s, *C*(CH_3_)_3_, dl and *meso*), 124.95 (q, *J*_CF_=275.1 Hz, CF_3_, dl or *meso*), 125.27 (q, *J*_CF_=275.0 Hz, CF_3_, dl or *meso*), 168.77 (s, C=O, dl or *meso*), 168.98 (s, C=O, dl or *meso*) ppm; HRMS (EI): *m*/*z*: [*M*+H]^+^ calcd for C_16_H_25_F_6_O_4_: 395.1652, found: 395.1649.

**Di-*tert*-butyl 2,3-bis(2,2-difluoroethyl)succinate (3 f)**: Following purification of the crude product using column chromatography (5:1 hexane/EtOAc), **3 f** was obtained as a colorless liquid (collection of 5 mL; yield: 0.400 g, 40 %): ^1^H NMR (400 MHz, CDCl_3_) *δ*=5.86 (tm, *J*_HF_=57.0 Hz, 2 H, CHF_2_, dl and *meso*), 2.90–2.89 (m, 1 H, dl and *meso*), 2.74–2.71 (m, 1 H, dl and *meso*), 2.30–2.14 (m, 2 H, CH_2_, dl and *meso*), 1.87–1.70 (m, 2 H, CH_2_, dl and *meso*), 1.40 (s, 18 H, C(CH_3_)_3_, dl or *meso*), 1.39 (s, 18 H, C(CH_3_)_3_, dl or *meso*) ppm; ^13^C NMR (100 MHz, CDCl_3_): *δ*=28.30 (s, C(*C*H_3_)_3_, dl or *meso*), 28.33 (s, C(*C*H_3_)_3_, dl or *meso*), 33.14 (t, *J*=22.1 Hz, CH_2_, dl or *meso*), 34.26 (t, *J*=22.0 Hz, CH_2_, dl or *meso*), 41.82 (q, *J*=7.3, 3.5 Hz, CH, dl or *meso*), 42.47 (q, *J*=6.4, 4.0 Hz, CH, dl or *meso*), 82.65 (s, *C*(CH_3_)_3_, dl or *meso*), 82.70 (s, *C*(CH_3_)_3_, dl or *meso*), 116.21 (t, *J*=238.0 Hz, CHF_2_, dl or *meso*), 116.43 (t, *J*=238.0 Hz, CHF_2_, dl or *meso*), 171.39 (s, C=O, dl and *meso*) ppm; HRMS (EI): *m*/*z*: [*M*+H]^+^ calcd for C_16_H_27_F_4_O_4_: 359.1840, found: 359.1839.

**Dimethyl 2,3-dimethyl-2,3-bis(2,2,2-trifluoroethyl)succinate (3 g)**: Following purification of the crude product using column chromatography (3:1 CH_2_Cl_2_/hexane), **3 g** was obtained as a white solid (collection of 5 mL; yield: 90 mg, 11 %): mp: 53.5–54.5 °C; ^1^H NMR (400 MHz, CDCl_3_): *δ*=3.67 (s, 6 H, dl or *meso*), 3.66 (s, 6 H, dl or *meso*), 3.00 (dq, *J*=16.4, 11.6 Hz, 2 H, dl and *meso*), 2.82–2.67 (m, 2 H, dl or *meso*), 2.14 (dq, *J*=15.1, 10.0 Hz, 2 H, dl or *meso*), 1.25 (s, 6 H, dl or *meso*), 1.16 (s, 6 H, dl or *meso*) ppm; ^13^C NMR (100 MHz, CDCl_3_): *δ*=17.01 (s, OCH_3_, dl or *meso*), 38.45 (q, *J*=27.8 Hz, CH_2_, dl or *meso*), 48.47 (s, CH, dl or *meso*), 49.24 (s, CH, dl or *meso*), 53.08 (s, CH_3_, dl and *meso*), 126.54 (q, *J*=276.1 Hz, CF_3_, dl or *meso*), 173.11 (s, C=O, dl or *meso*), 173.34 (s, C=O, dl or *meso*) ppm; HRMS (APCI): *m*/*z*: [*M*+H]^+^ calcd for C_12_H_17_F_6_O_4_: 339.1026, found: 339.1025.

**Dimethyl 2,3-bis(2,2-difluoroethyl)-2,3-dimethylsuccinate (3 h)**: Following purification of the crude product using column chromatography (3:1 CH_2_Cl_2_/hexane), **3 h** was obtained as a colorless oil (collection of 5 mL; yield: 0.123 mg, 16 %): ^1^H NMR (400 MHz, CDCl_3_): *δ*=5.75 (tm, *J*_HF_=56.0 Hz, 2 H, CHF_2_, dl and *meso*), 3.65 (s, 6 H, dl or *meso*), 3.64 (s, 6 H, dl or *meso*), 2.66–2.43 (m, 2 H, dl and *meso*), 2.37–2.24 (m, 2 H, dl or *meso*), 1.91–1.78 (m, 2 H, dl or *meso*), 1.22 (m, 6 H, dl or *meso*), 1.14 (m, 6 H, dl or *meso*) ppm; ^13^C NMR (100 MHz, CDCl_3_): *δ*=17.78 (s, CH_3_, dl or *meso*), 18.01 (s, CH_3_, dl or *meso*), 38.26 (t, *J*=22.1 Hz, CH, dl or *meso*), 38.71 (t, *J*=21.9 Hz, CH, dl or *meso*), 48.84 (dd, *J*=9.4, 3.5 Hz, CH_2_, dl or *meso*), 49.45 (t, *J*=4.7 Hz, CH_2_, dl or *meso*), 52.85 (s, CH_3_, dl and *meso*), 116.60 (t, *J*_CF_=238.1 Hz, CHF_2_, dl or *meso*), 116.72 (t, *J*_CF_=238.1 Hz, CHF_2_, dl or *meso*), 173.90 (s, C=O, dl or *meso*), 174.02 (s, C=O, dl or *meso*) ppm; HRMS (ESI): *m*/*z*: [*M*+H]^+^ calcd for C_12_H_19_F_4_O_4_: 303.1217, found: 303.1214.

**Electrolchemical di-and trifluoromethyl acetamidation**: A mixture of **1 a** or **1 b** (6 mmol), **2 d** (2 mmol, 0.20 g), and Et_3_N (0.06 mmol, 61 mg) dissolved in CH_3_CN/H_2_O (7:1 *v*/*v*, 10 mL) was introduced into the electrochemical microreactor equipped with a large FEP channel (Figure 1, right) through a syringe pump (5 μL min^−1^; residence time: 636 s) with an applied current of 10 mA (current density: 2.4 mA cm^−2^) and collected in a glass vial at the outlet. The reaction solution was neutralized with saturated aq NaHCO_3_ (4 mL), the organic layer was separated, and the aqueous layer was extracted with EtOAc (3×10 mL). The combined organic layers were washed with brine (20 mL), dried over MgSO_4_, and concentrated in vacuo. The residual yellow oil was purified by column chromatography (silica gel) equilibrated with a mixture of hexane and EtOAc as eluent. The yields of **4 a** and **4 b** were determined on the basis of **2 d**.

**Methyl 2-(acetylamino)-2-methyl-4,4,4-trifluorobutyrate (4 a)**:[10a] Following purification of the crude product using column chromatography (2:1 hexane/EtOAc), **4 a** was obtained as a white solid (collection of 10 mL; yield: 0.111 g, 25 %): mp: 94.0–96.3 °C; ^1^H NMR (400 MHz, CDCl_3_): *δ*=6.51 (s, 1 H), 3.74 (s, 3 H), 3.30 (dq, 1 H, *J*=15.7, 10.7 Hz, 1 H), 2.78 (dq, 1 H, *J*=15.7, 10.8 Hz, 1 H), 1.93 (s, 3 H), 1.59 (s, 3 H) ppm; ^13^C NMR (100 MHz, CDCl_3_): *δ*=22.7 (s, CH_3_), 22.8 (s, CH_3_), 36.6 (q, *J*=27.0 Hz, CH_2_), 52.4 (s, OCH_3_), 55.3 (q, *J*=2.3 Hz, CNH), 124.6 (q, *J*_CF_=276.0 Hz, CF_3_), 169.1 (s, C=O), 172.3 (s, C=O) ppm; HRMS (EI): *m*/*z*: [*M*+H]^+^ calcd for C_8_H_13_F_3_NO_3_: 228.0842, found: 228.0841.

**Methyl 2-(acetylamino)-2-methyl-4,4-difluorobutyrate (4 b)**: Following purification of the crude product using column chromatography (1:1 hexane/EtOAc), **4 b** was obtained as a white solid (collection of 10 mL; yield: 0.113 g, 27 %): mp: 56.2–57.2 °C; ^1^H NMR (CDCl_3_): *δ*=6.74 (s, 1 H), 5.78 (tdd, *J*=28.0, 5.2, 3.6 Hz, 1 H), 3.71 (s, 3 H), 2.86–2.72 (m, 1 H), 2.54–2.42 (m, 1 H, CH_2_), 1.94 (s, 3 H), 1.52 (s, 3 H) ppm; ^13^C NMR (100 MHz, CDCl_3_): *δ*=23.7 (s, CH_3_), 24.0 (s, CH_3_), 39.5 (t, *J*=10.8 Hz, CH_2_), 53.2 (s, OCH_3_), 56.8 (t, *J*=3.1 Hz, CNH), 115.9 (t, *J*_CF_=118.5 Hz, CHF_2_), 170.3 (s, C=O), 174.0 (s, C=O) ppm; HRMS (EI): *m*/*z*): [*M*+H]^+^ calcd for C_8_H_14_F_2_NO_3_: 210.0936, found: 210.0935.

**Electrochemical bis(trifluoromethylation)**: A mixture of **1 a** or **1 b** (16 mmol), **2** (1 mmol), and Et_3_N (0.16 mmol) dissolved in CH_3_CN/H_2_O (7:1 *v*/*v*, 10 mL) was introduced into the electrochemical microreactor equipped with a large FEP channel (Figure 1, left) through a syringe pump (50 μL min^−1^; residence time: 28 s) with an applied current of 200 mA (current density: 111 mA cm^−2^) and collected in a glass vial at the outlet. The reaction solution was neutralized with saturated aq NaHCO_3_ (6 mL), the organic layer was separated, and the aqueous layer was extracted with EtOAc (3×10 mL). The combined organic layers were washed with brine (20 mL), dried over MgSO_4_, and concentrated in vacuo. The residual yellow oil was purified by column chromatograph (silica gel) equilibrated with a mixture of hexane and EtOAc as eluent. The yields of **6** were determined on the basis of the used alkene.

**4,4,4-Trifluoro-2-(trifluoromethyl)butanamide (6 a)**:[10c] Following purification of the crude product using column chromatography (1:1 hexane/EtOAc), **6 a** was obtained as a colorless solid (collection of 10 mL; yield: 0.141 g, 67 %): mp: 89.6–93.0 °C; ^1^H NMR (400 MHz, CD_3_OD): *δ*=8.18 (s, 1 H), 7.51 (d, 1 H), 3.63–3.54 (m, 1 H, CH), 3.08–2.93 (m, 1 H, CH_2_), 2.68–2.56 (m, 1 H, CH_2_) ppm; ^13^C NMR (100 MHz, CD_3_OD): *δ*=30.04 (q, *J*=30.7 Hz, CH_2_), 44.16 (q, *J*=27.3 Hz, CH_2_), 124.57 (q, *J*=277.5 Hz, CF_3_), 125.86 (q, *J*=273.8 Hz, CF_3_), 167.44 (s, C=O) ppm; MS (APCI): *m*/*z* (%): 210.03 (100) [*M*+H]^+^.

**2-(Difluoromethyl)-4,4-difluorobutanamide (6 b)**: Following purification of the crude product using column chromatography (4:1 Et_2_O/EtOAc), **6 b** was obtained as a colorless solid (collection of 10 mL; yield: 78 mg; 45 %): mp: 73.1–74.2 °C; ^1^H NMR (400 MHz, CD_3_OD) *δ*=7.61 (s, 1 H), 6.86 (s, 1 H), 5.62 (tm, *J*_HF_=55.8 Hz), 2.74–2.63 (m, 1 H), 2.10–1.94 (m, 1 H), 1.83–1.69 (m, 1 H) ppm; ^13^C NMR (100 MHz, CD_3_OD): *δ*=31.92 (tm, *J*=22.7 Hz, CH_2_), 46.16 (tm, *J*=21.7, CH_2_), 117.37 (t, *J*=236.6 Hz, CHF_2_), 117.81 (t, *J*=241.5 Hz, CHF_2_), 164.94 (s, C=O) ppm; HRMS (APCI): *m*/*z*: [*M*+H]^+^ calcd for C_5_H_8_F_4_NO: 174.0537, found: 174.0534.

**2-(Difluoromethyl)-4,4-difluoro-*N*-methylbutanamide (6 c)**: Following purification of the crude product using column chromatography (3:1 hexane/EtOAc), **6 b** was obtained as a colorless oil (collection of 10 mL; yield: 61 mg, 32 %): ^1^H NMR (400 MHz, CDCl_3_): *δ*=9.21 (s, 1 H), 5.92 (tm, *J*_HF_=55.6 Hz, 2 H), 3.70 (br, 1 H, CH), 3.13 (s, 3 H), 2.62–2.46 (m, 1 H), 2.29–2.14 (m, 1 H) ppm; ^13^C NMR (100 MHz, CDCl_3_): *δ*=27.98 (s, CH_3_), 31.88 (t, *J*=21.1 Hz, CH_2_), 42.75 (t, *J*=22.4 Hz, CH), 114.82 (t, *J*_CF_=159.0 Hz, CHF_2_), 115.90 (t, *J*_CF_=162.63 Hz, CHF_2_), 162.06 (s, C=O) ppm; HRMS (EI): *m*/*z*: [*M*+H]^+^ calcd for C_6_H_10_F_4_NO: 188.0693, found: 188.0692.
