# Supplementary material for: Difluoro-and Trifluoromethylation of Electron-Deficient Alkenes in an Electrochemical Microreactor
Source: ChemistryOpen. 2013 Nov 26;3(1):23–8. doi: 10.1002/open.201300039 (PMC3943609; doi:10.1002/open.201300039)
Supplement: Supplementary file 1 [file open0003-0023-sd1.pdf]

ChemistryOPEN

Supporting Information

© 2013 The Authors. Published by Wiley-VCH Verlag GmbH & Co. KGaA, Weinheim

Difluoro- and Trifluoromethylation of Electron-Deficient Alkenes in an Electrochemical Microreactor

Kenta Arai, Kevin Watts, and Thomas Wirth*[a]

open_201300039_sm_miscellaneous_information.pdf

Contents

S1: Contents

S2: Figure S1: Electrochemical microreactor

S3-S15: ^{1}H and ^{13}C NMR spectra

- S3: Dimethyl 2,3-bis(2,2,2-trifluoroethyl)succinate (**3a**)
- S4: Dimethyl 2,3-bis(2,2-difluoroethyl)succinate (**3b**)
- S5: Diethyl 2,3-bis(2,2,2-trifluoroethyl)succinate (**3c**)
- S6: Diethyl 2,3-bis(2,2-difluoroethyl)succinate (**3d**)
- S7: Di-*tert*-butyl 2,3-bis(2,2,2-trifluoroethyl)succinate (**3e**)
- S8: Di-*tert*-butyl 2,3-bis(2,2-difluoroethyl)succinate (**3f**)
- S9: Dimethyl 2,3-dimethyl-2,3-bis(2,2,2-trifluoroethyl)succinate (**3g**)
- S10: Dimethyl 2,3-bis(2,2-difluoroethyl)-2,3-dimethylsuccinate (**3h**)
- S11: Methyl 2-(acetylamino)-2-methyl-4,4,4-trifluorobutyrate (**4a**)
- S12: Methyl 2-(acetylamino)-2-methyl-4,4-difluorobutyrate (**4b**)
- S13: 4,4,4-Trifluoro-2-(trifluoromethyl)butanamide (**6a**)
- S14: 2-(Difluoromethyl)-4,4-difluorobutanamide (**6b**)
- S15: 2-(Difluoromethyl)-4,4-difluoro-*N*-methylbutanamide (**6c**)

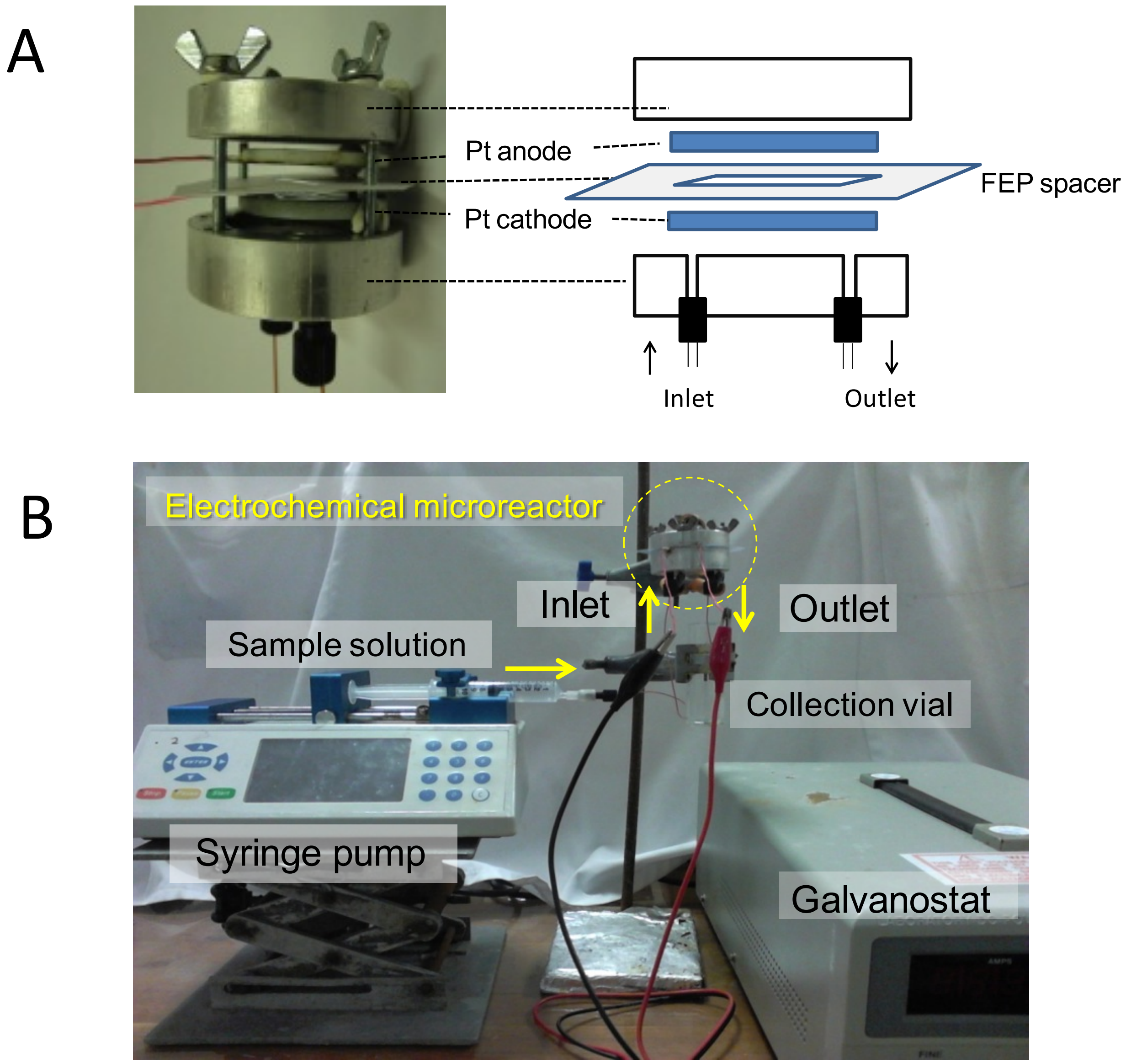

Figure S1. Electrochemical microreactor. (A) An exploded photograph of the device and a schematic representation. (B) Photograph of the general microreactor set-up

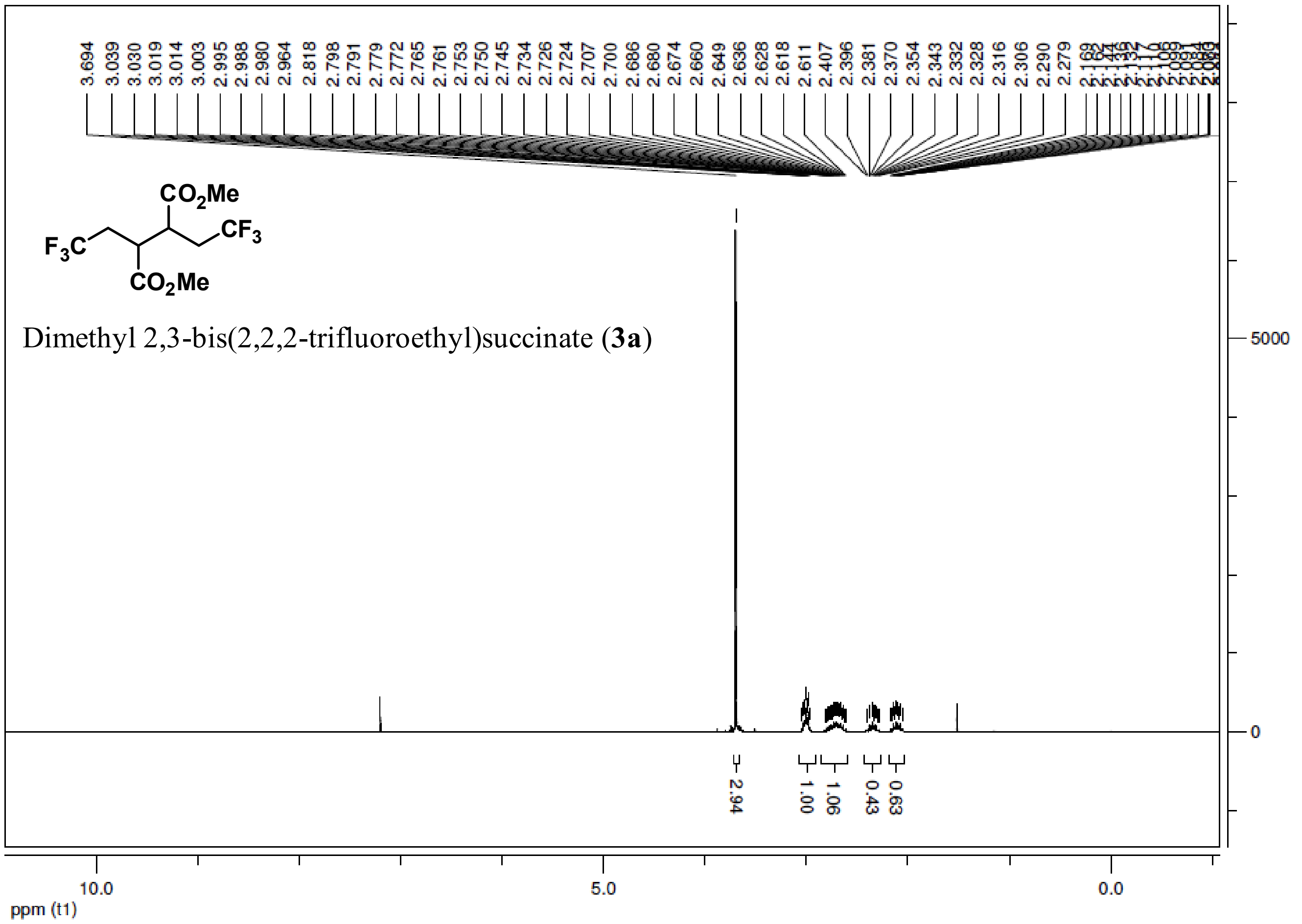
Dimethyl 2,3-bis(2,2,2-trifluoroethyl)succinate (**3a**)
2.94
1.00
1.06
0.43
0.63
ppm (t1)

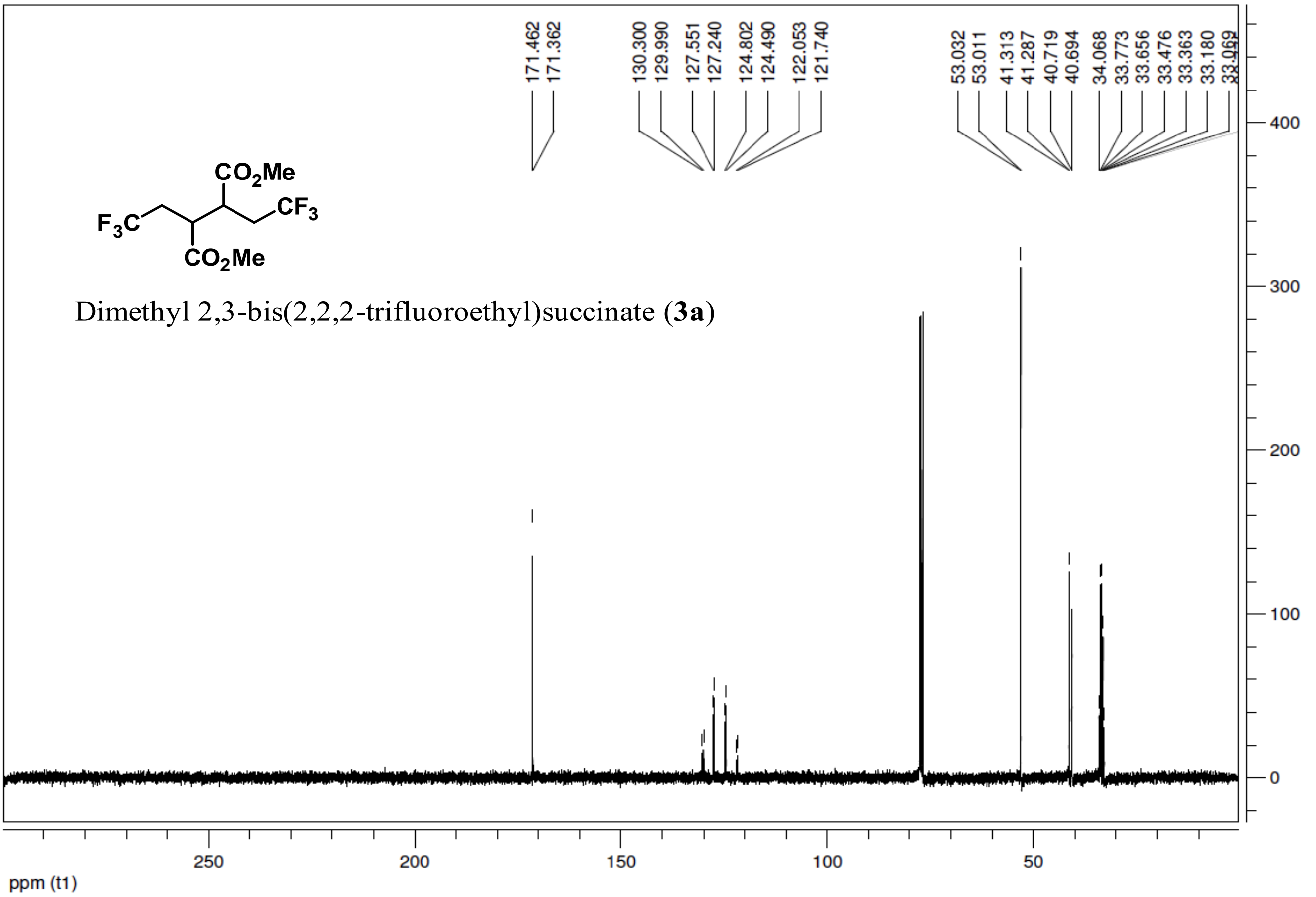
Dimethyl 2,3-bis(2,2,2-trifluoroethyl)succinate (**3a**)
ppm (t1)

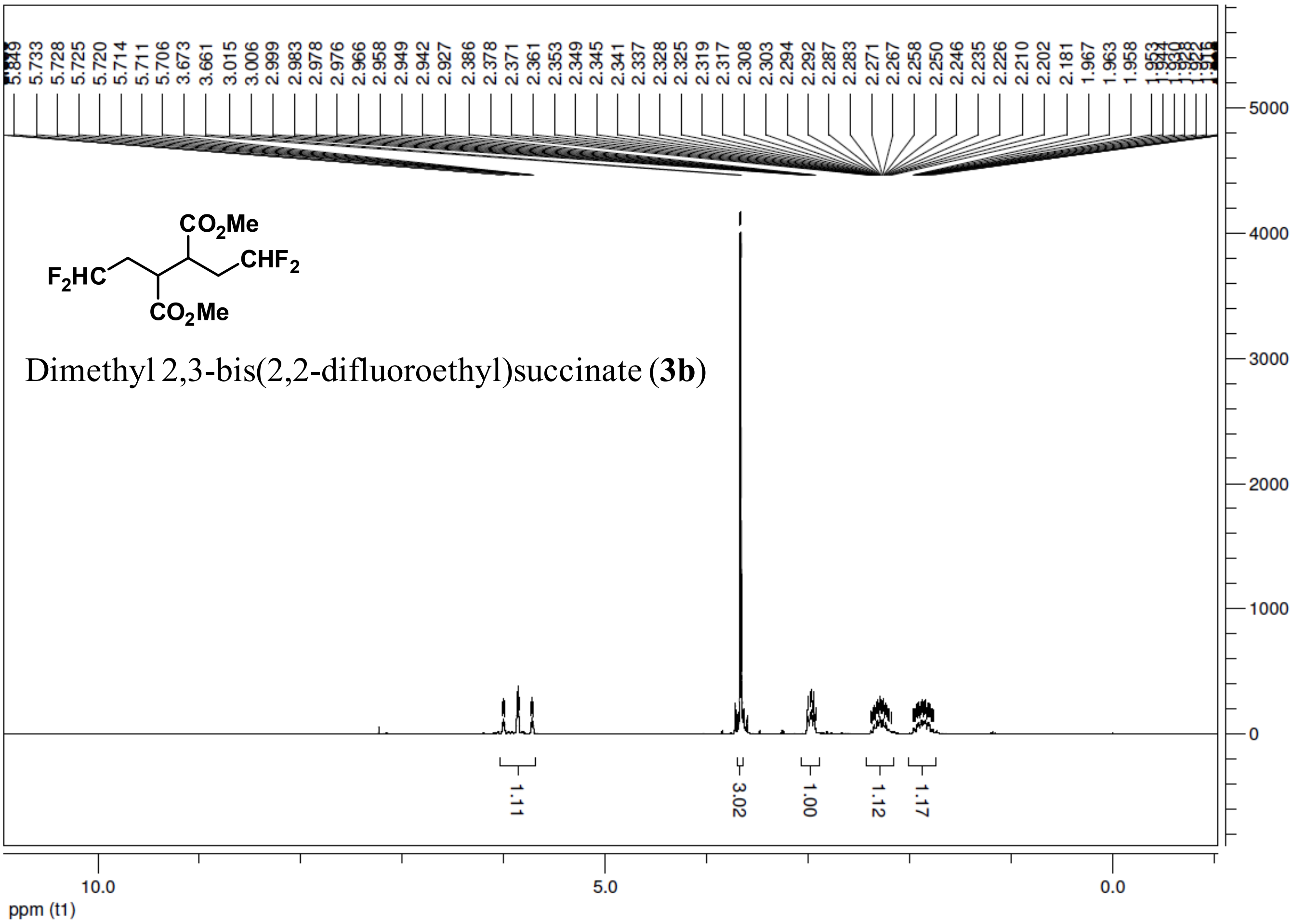

CO2Me
F2HC
CHF2
CO2Me
Dimethyl 2,3-bis(2,2-difluoroethyl)succinate (3b)
5.733
5.728
5.725
5.720
5.714
5.711
5.706
3.673
3.661
3.015
3.006
2.999
2.983
2.978
2.976
2.966
2.958
2.949
2.942
2.927
2.386
2.378
2.371
2.361
2.353
2.349
2.345
2.341
2.337
2.328
2.325
2.319
2.317
2.308
2.303
2.294
2.292
2.287
2.283
2.271
2.267
2.258
2.250
2.246
2.235
2.226
2.210
2.202
2.181
1.967
1.963
1.958
1.11
3.02
1.00
1.12
1.17
5000
4000
3000
2000
1000
0
10.0
5.0
0.0
ppm (t1)

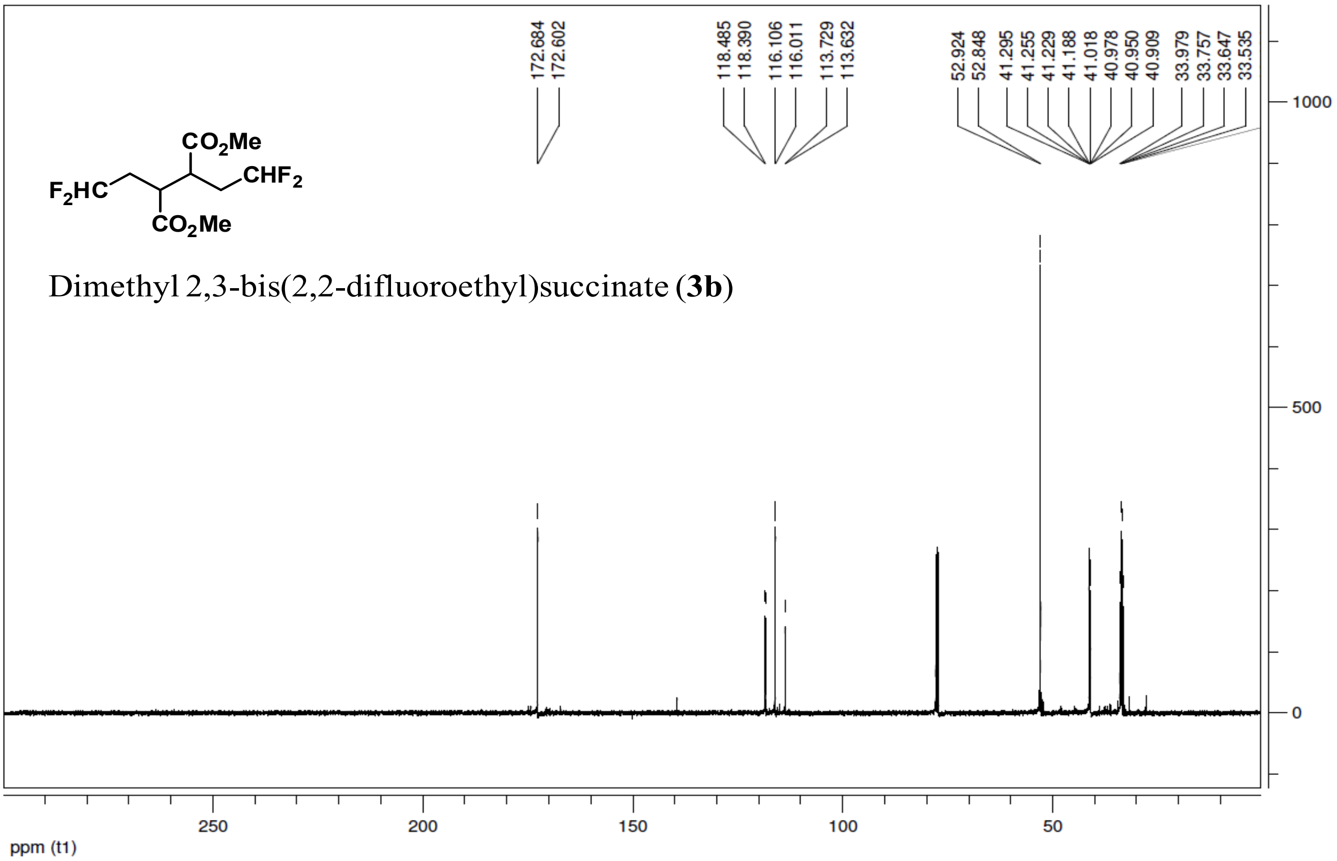

172.684
172.602
118.485
118.390
116.106
116.011
113.729
113.632
52.924
52.848
41.295
41.255
41.229
41.188
41.018
40.978
40.950
40.909
33.979
33.757
33.647
33.535
CO2Me
F2HC
CHF2
CO2Me
Dimethyl 2,3-bis(2,2-difluoroethyl)succinate (3b)
1000
500
0
250
200
150
100
50
ppm (t1)

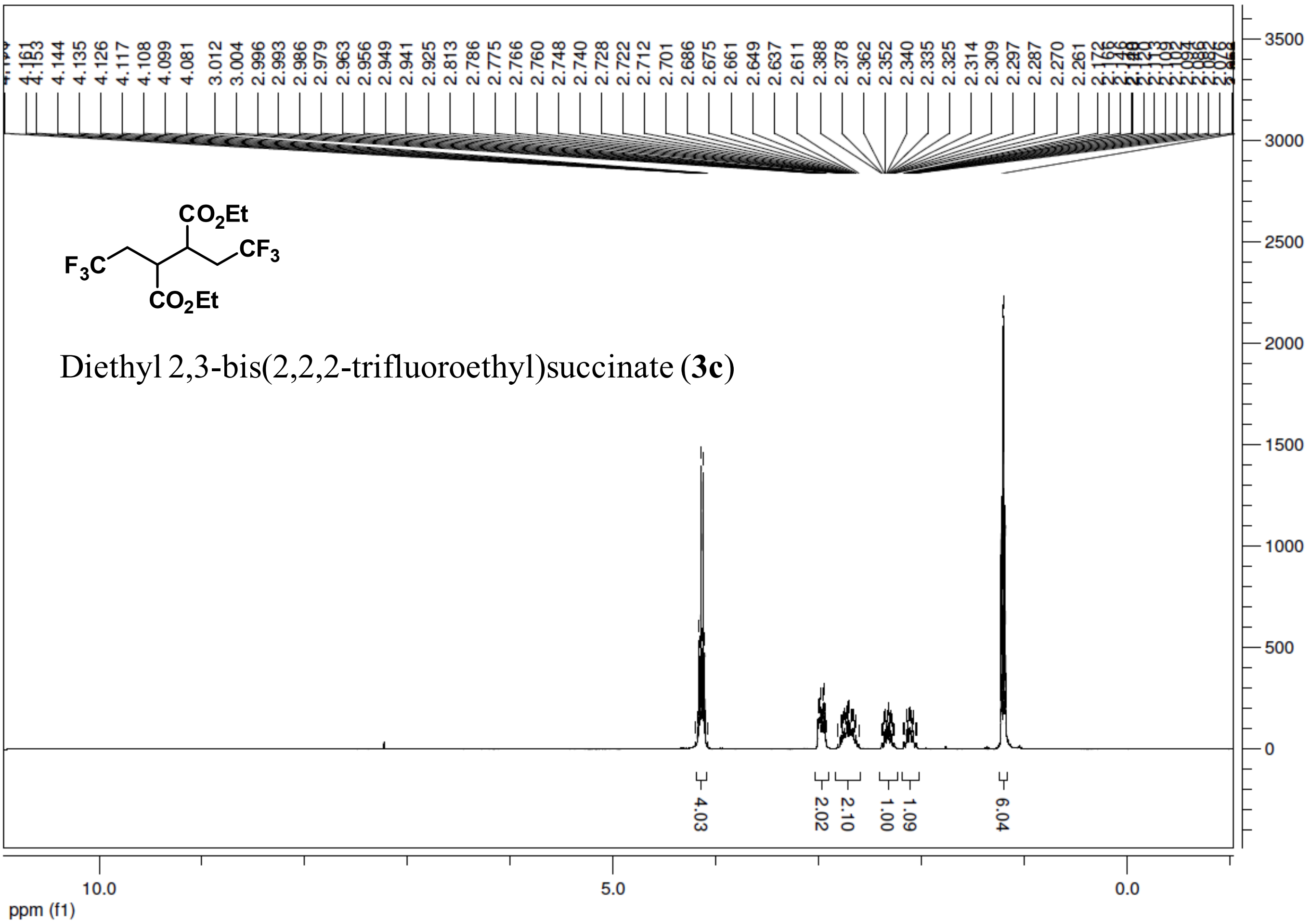

Diethyl 2,3-bis(2,2,2-trifluoroethyl)succinate (**3c**)

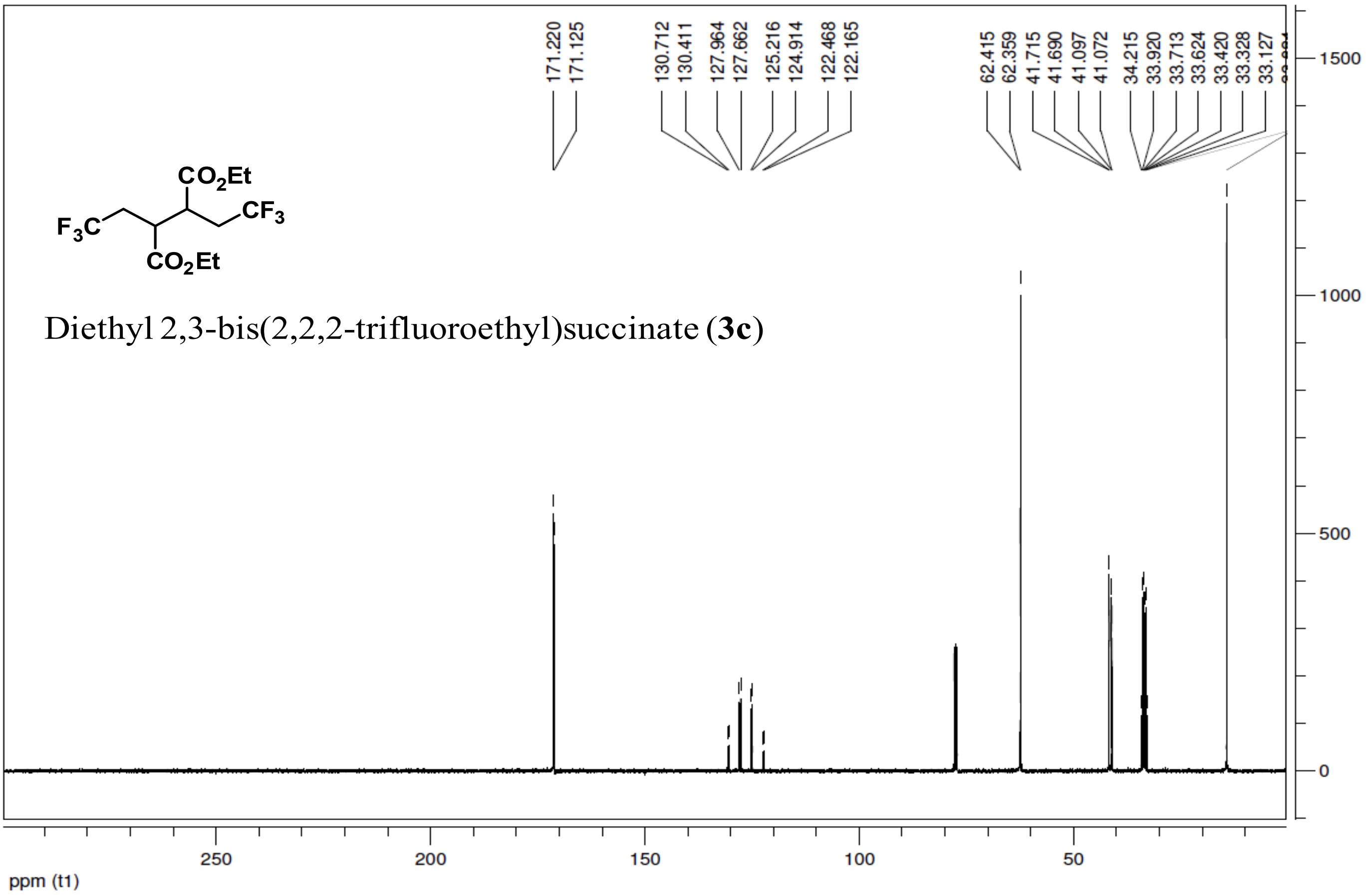

Diethyl 2,3-bis(2,2,2-trifluoroethyl)succinate (**3c**)

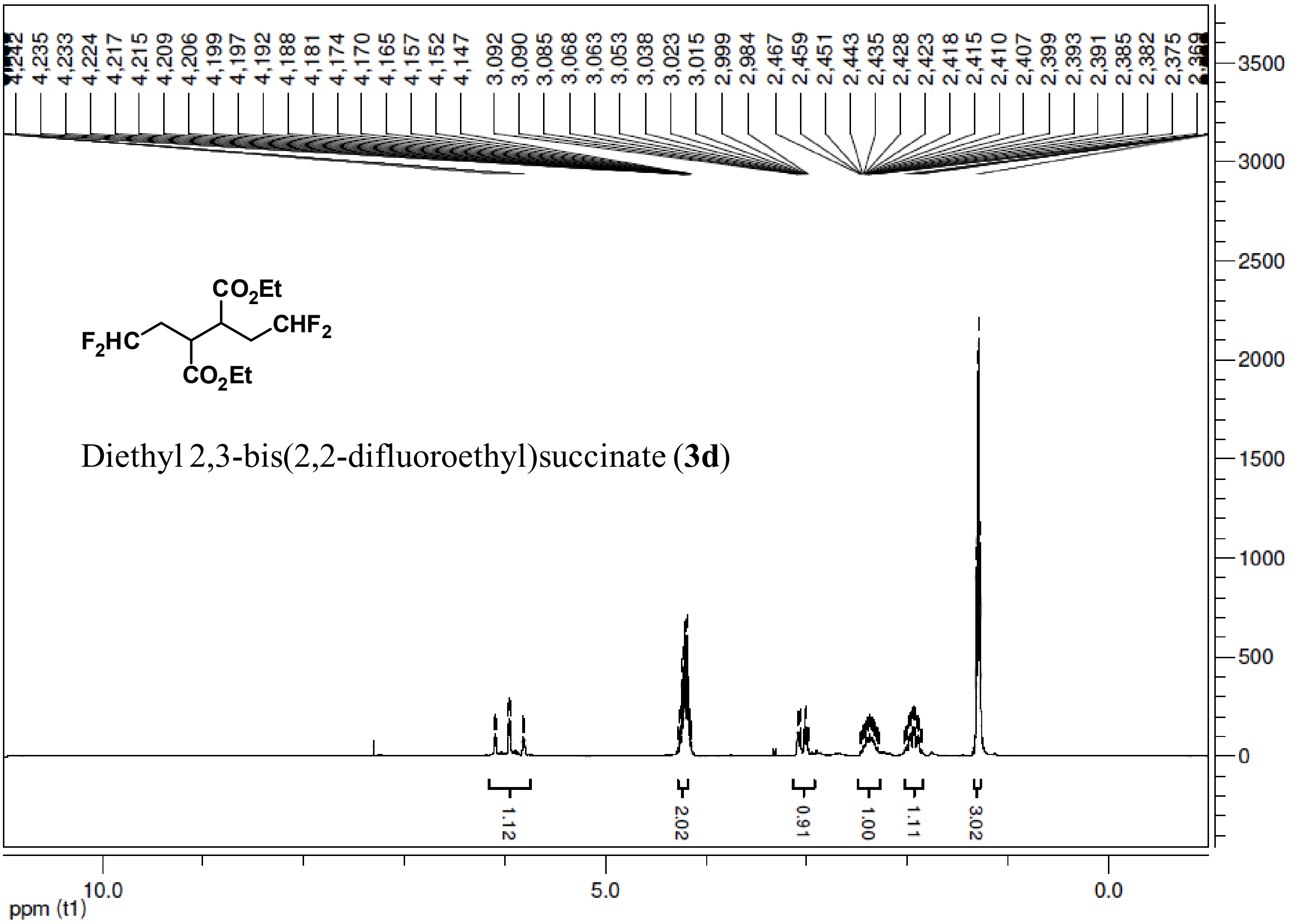
4,235 4,233 4,224 4,217 4,215 4,209 4,206 4,199 4,197 4,192 4,188 4,181 4,174 4,170 4,165 4,157 4,152 4,147 3,092 3,090 3,085 3,068 3,063 3,053 3,038 3,023 3,015 2,999 2,984 2,467 2,459 2,451 2,443 2,435 2,428 2,423 2,418 2,415 2,410 2,407 2,399 2,393 2,391 2,385 2,382 2,375
CO2Et
F2HC
CHF2
CO2Et
Diethyl 2,3-bis(2,2-difluoroethyl)succinate (3d)
1.12
2.02
0.91
1.00
1.11
3.02
3500
3000
2500
2000
1500
1000
500
0
10.0
5.0
0.0
ppm (t1)

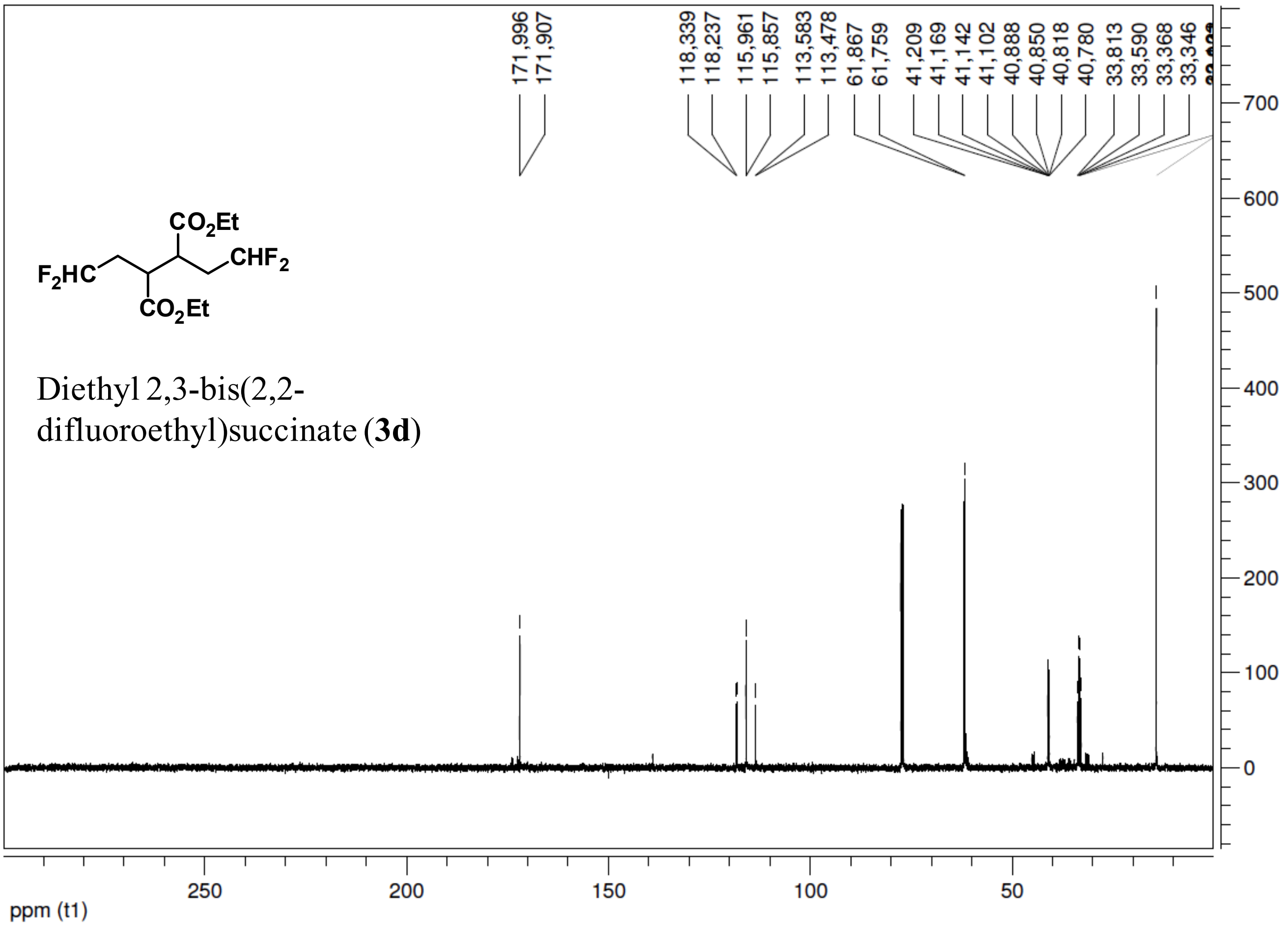
171,996 171,907 118,339 118,237 115,961 115,857 113,583 113,478 61,867 61,759 41,209 41,169 41,142 41,102 40,888 40,850 40,818 40,780 33,813 33,590 33,368 33,346
CO2Et
F2HC
CHF2
CO2Et
Diethyl 2,3-bis(2,2-difluoroethyl)succinate (3d)
700
600
500
400
300
200
100
0
250
200
150
100
50
ppm (t1)

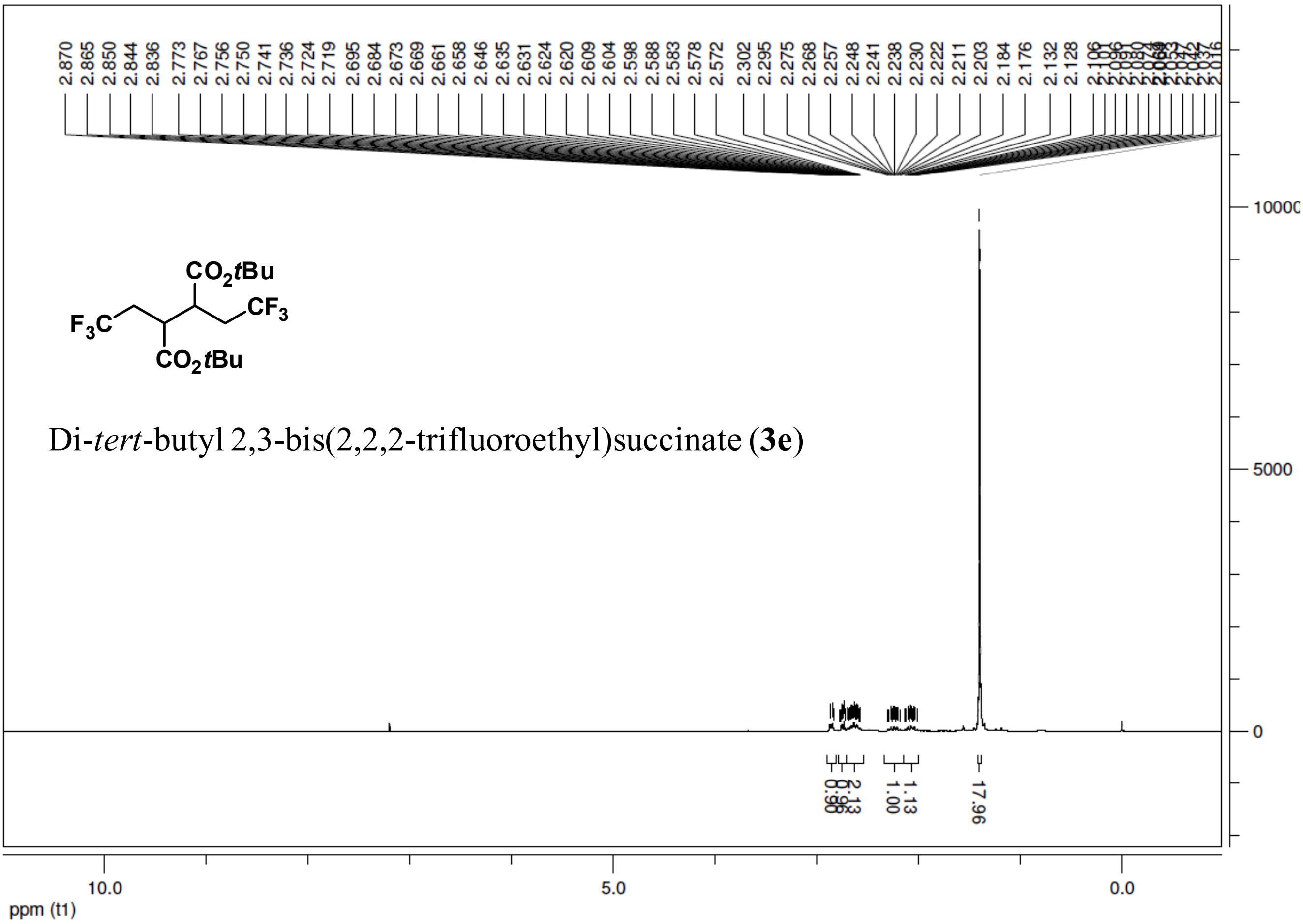

Di-*tert*-butyl 2,3-bis(2,2,2-trifluoroethyl)succinate (**3e**)

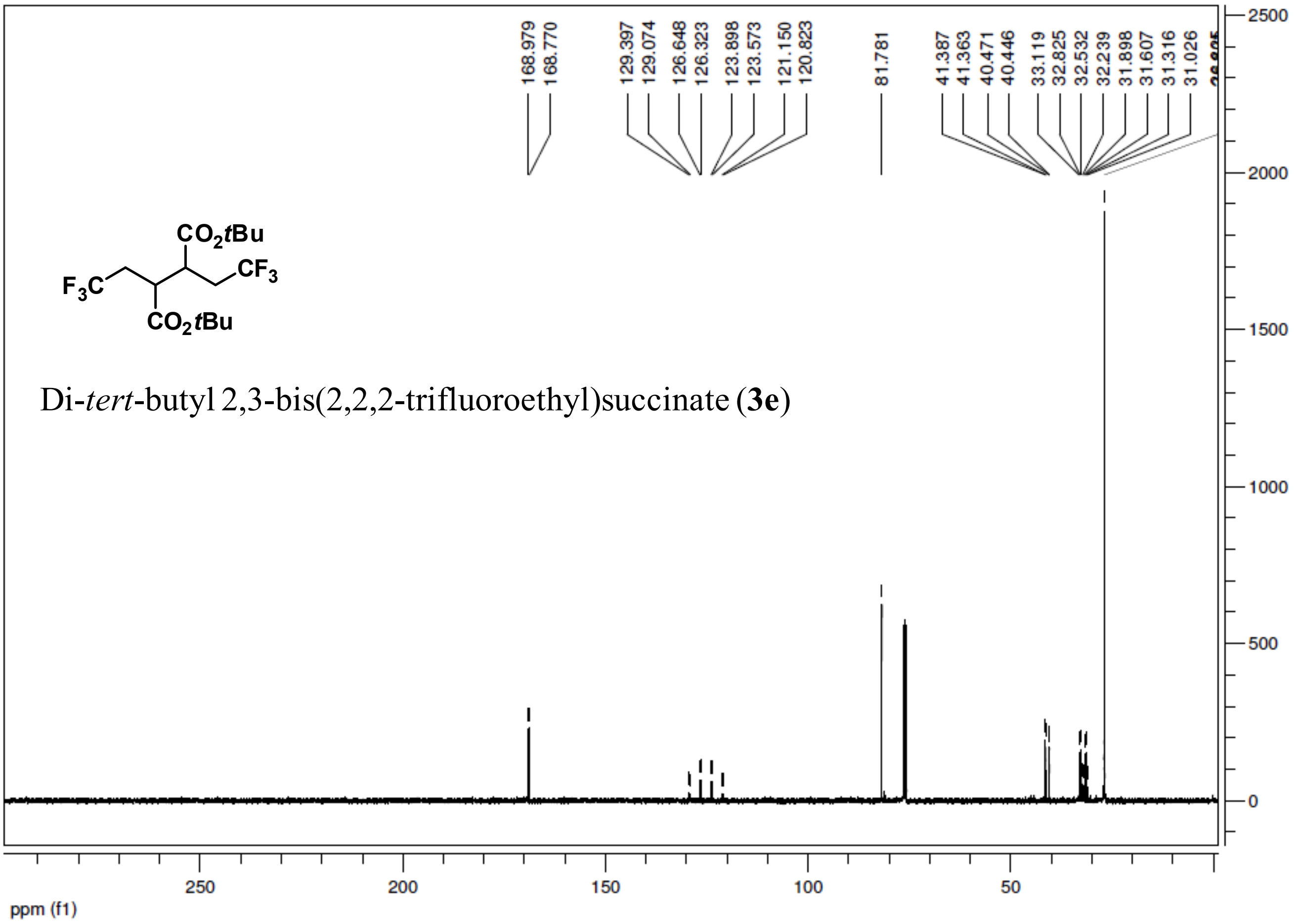

Di-*tert*-butyl 2,3-bis(2,2,2-trifluoroethyl)succinate (**3e**)

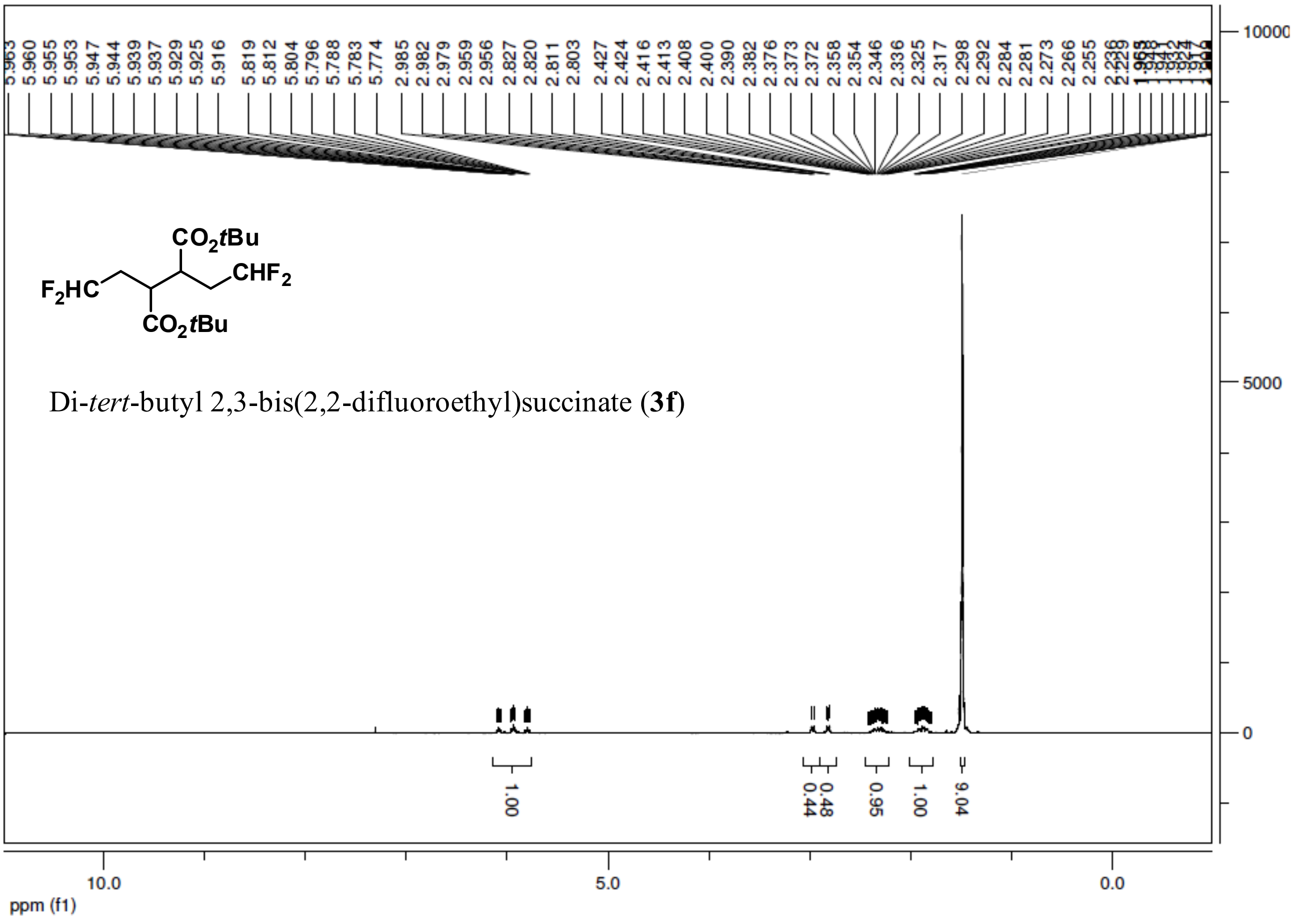

Di-*tert*-butyl 2,3-bis(2,2-difluoroethyl)succinate (**3f**)
1.00
0.44
0.48
0.95
1.00
9.04
ppm (f1)

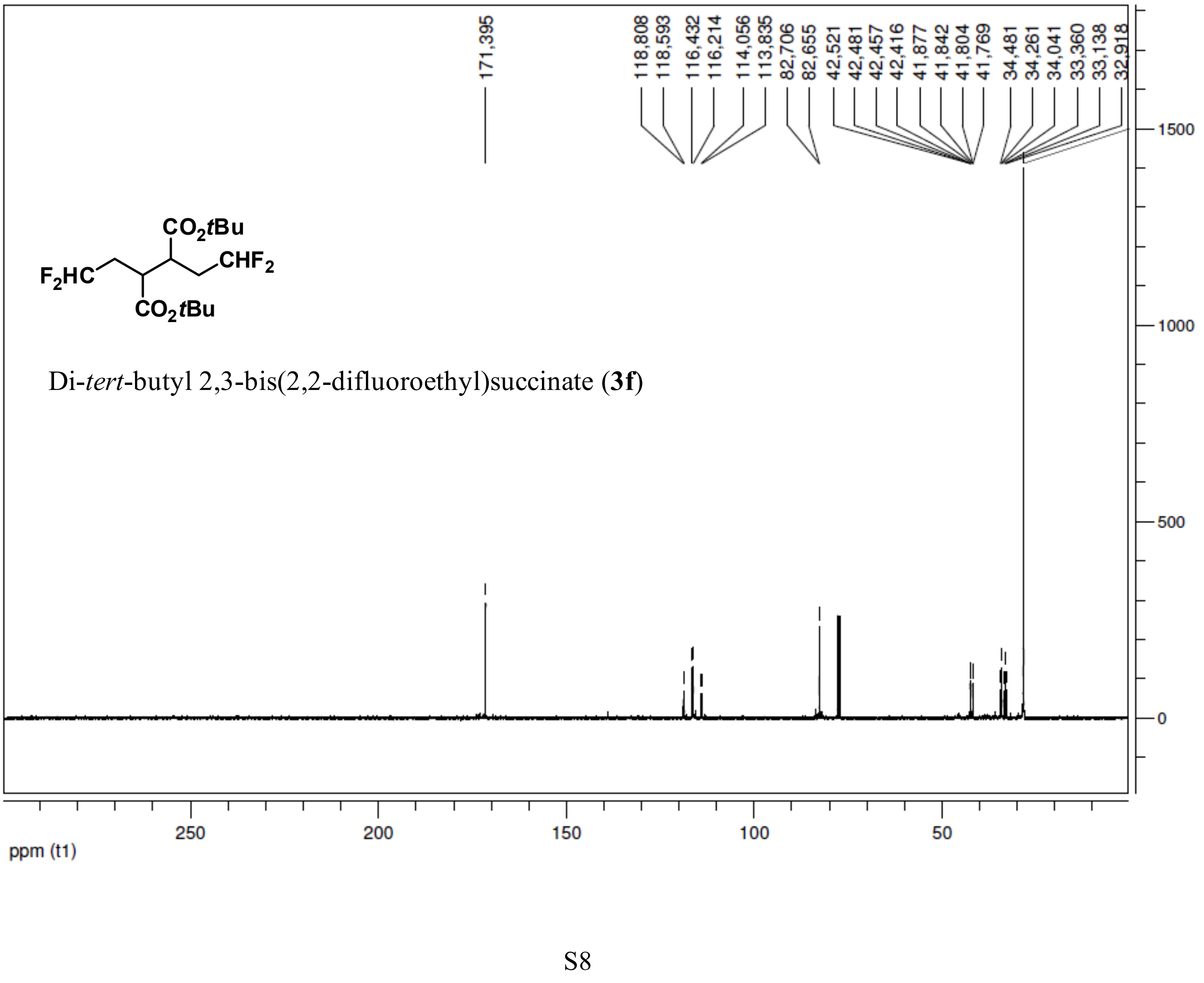

Di-*tert*-butyl 2,3-bis(2,2-difluoroethyl)succinate (**3f**)
ppm (t1)

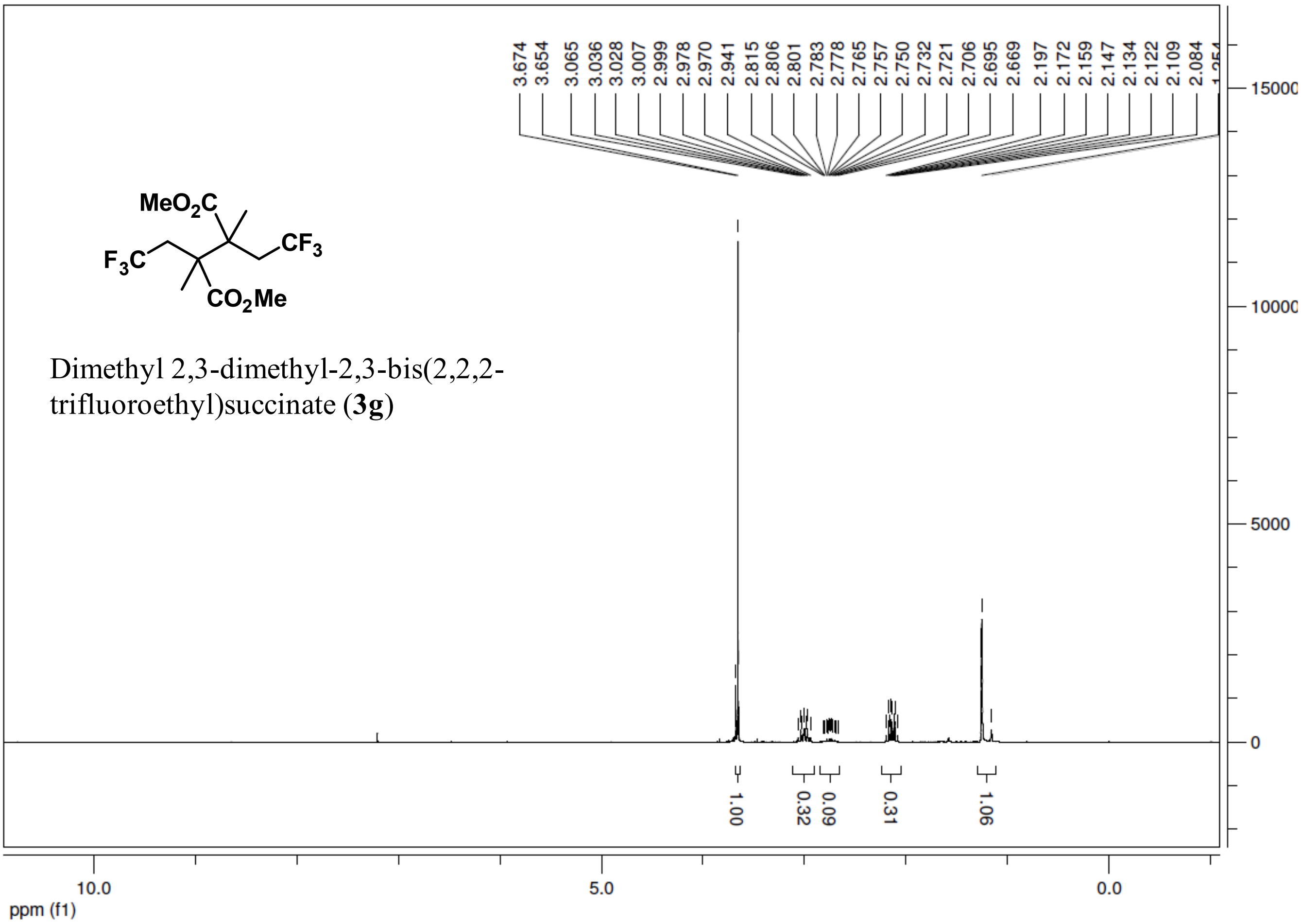
3.674 3.654 3.065 3.036 3.028 3.007 2.999 2.978 2.970 2.941 2.815 2.806 2.801 2.783 2.778 2.765 2.757 2.750 2.732 2.721 2.706 2.695 2.669 2.197 2.172 2.159 2.147 2.134 2.122 2.109 2.084
MeO2C
CF3
F3C
CO2Me
Dimethyl 2,3-dimethyl-2,3-bis(2,2,2-trifluoroethyl)succinate (3g)
1.00 0.32 0.09 0.31 1.06
15000
10000
5000
0
10.0
5.0
0.0
ppm (f1)

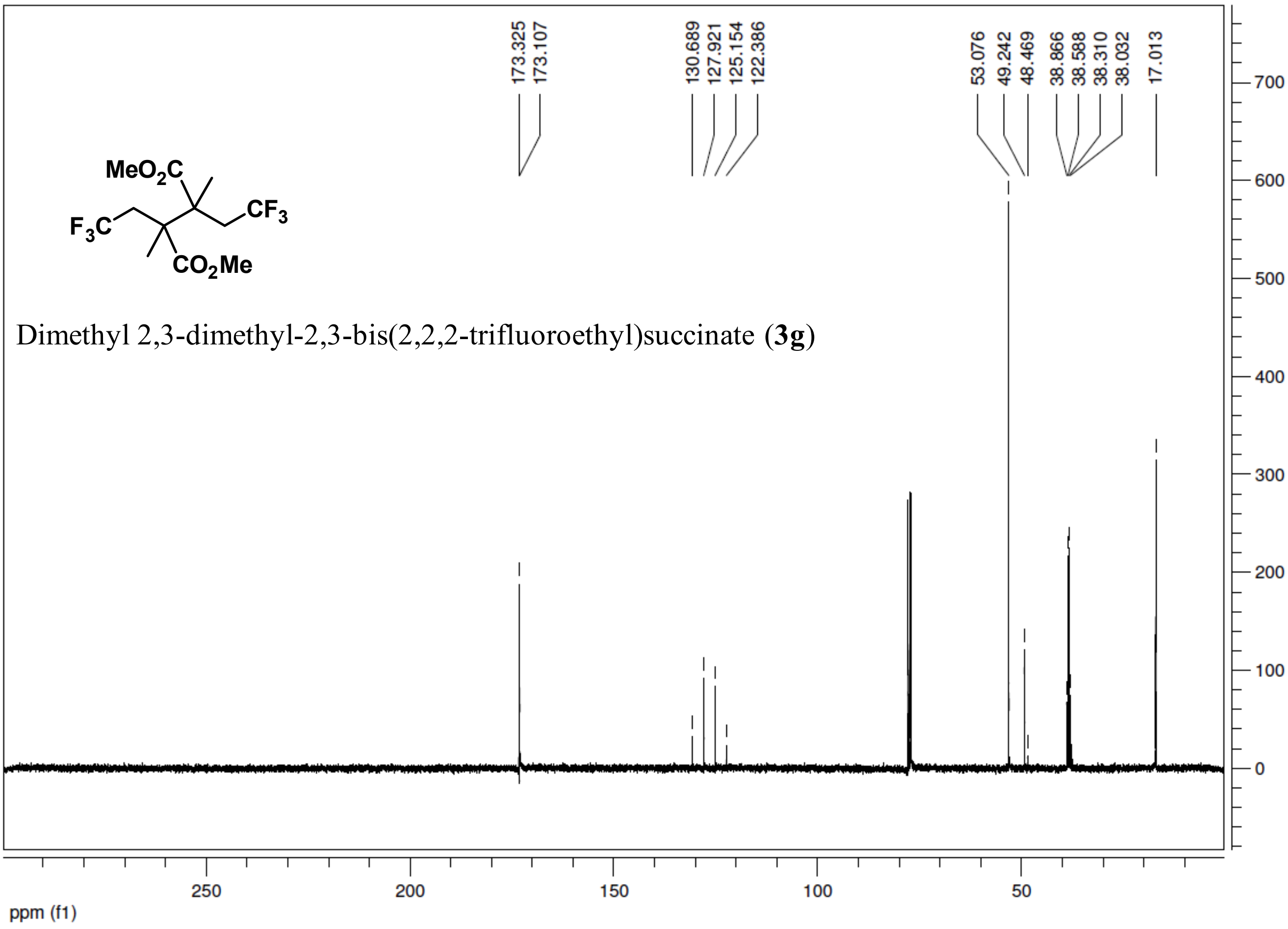
173.325 173.107
130.689 127.921 125.154 122.386
53.076 49.242 48.469 38.866 38.588 38.310 38.032 17.013
MeO2C
CF3
F3C
CO2Me
Dimethyl 2,3-dimethyl-2,3-bis(2,2,2-trifluoroethyl)succinate (3g)
700
600
500
400
300
200
100
0
250
200
150
100
50
ppm (f1)

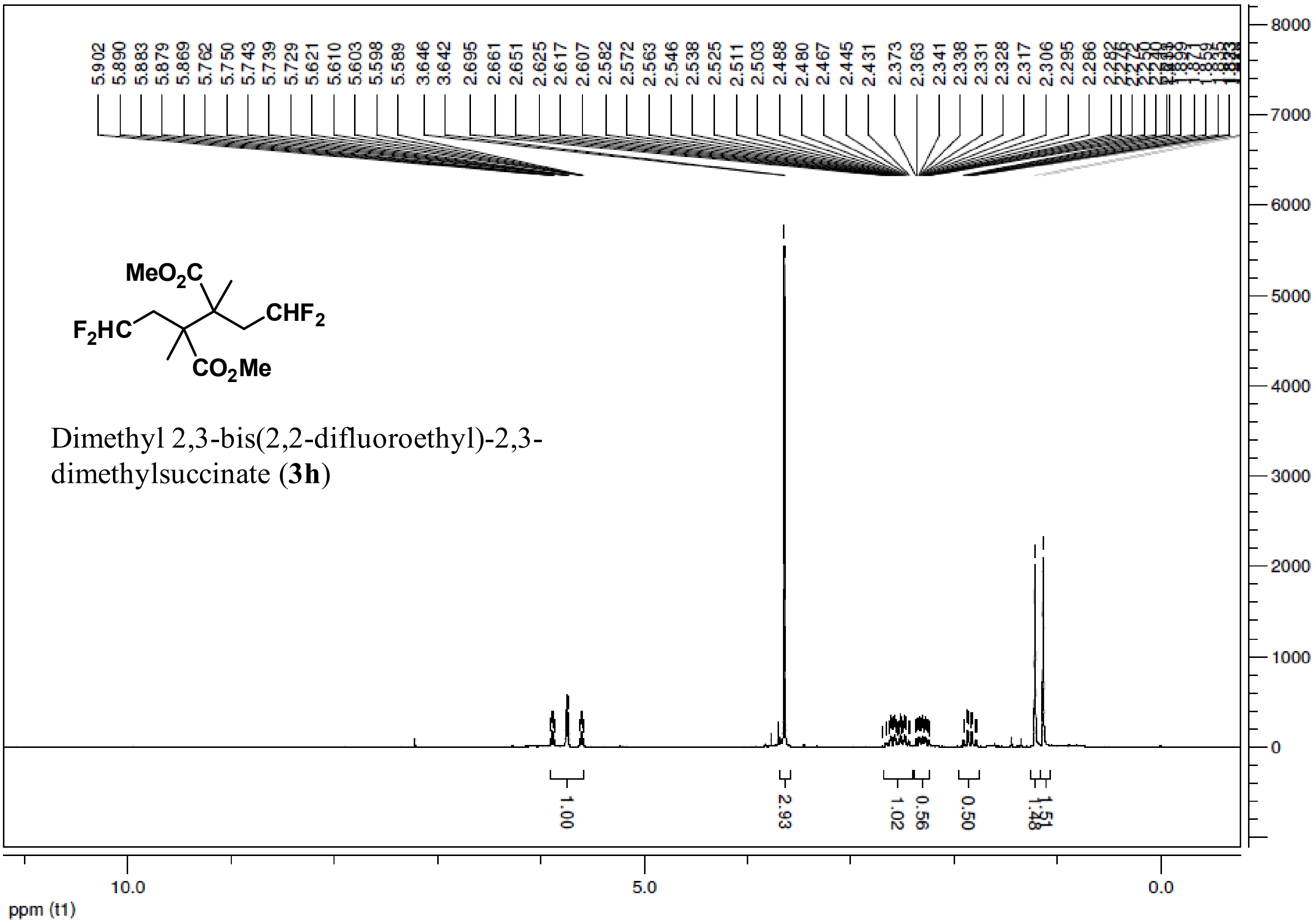

Dimethyl 2,3-bis(2,2-difluoroethyl)-2,3-dimethylsuccinate (**3h**)

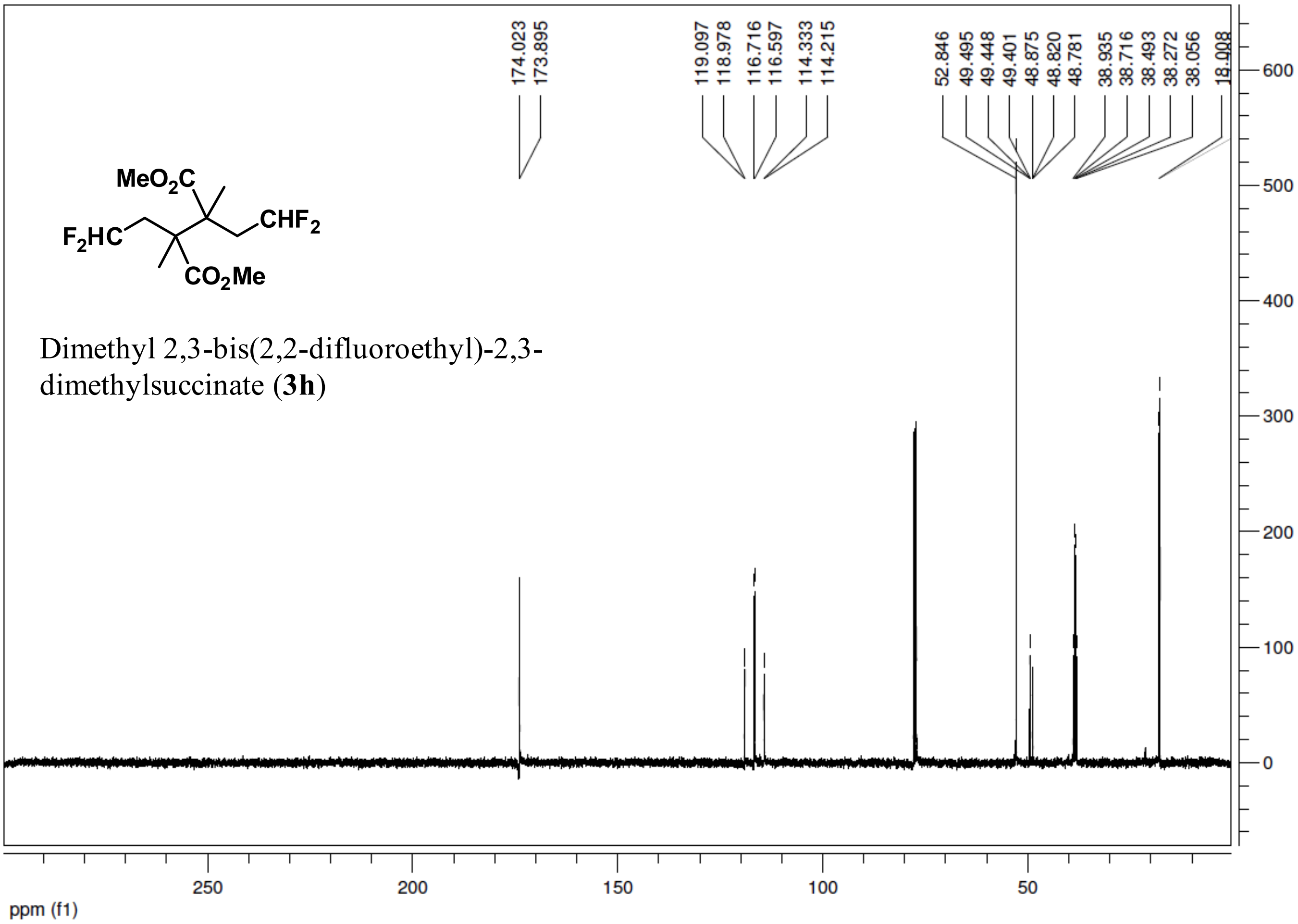

Dimethyl 2,3-bis(2,2-difluoroethyl)-2,3-dimethylsuccinate (**3h**)

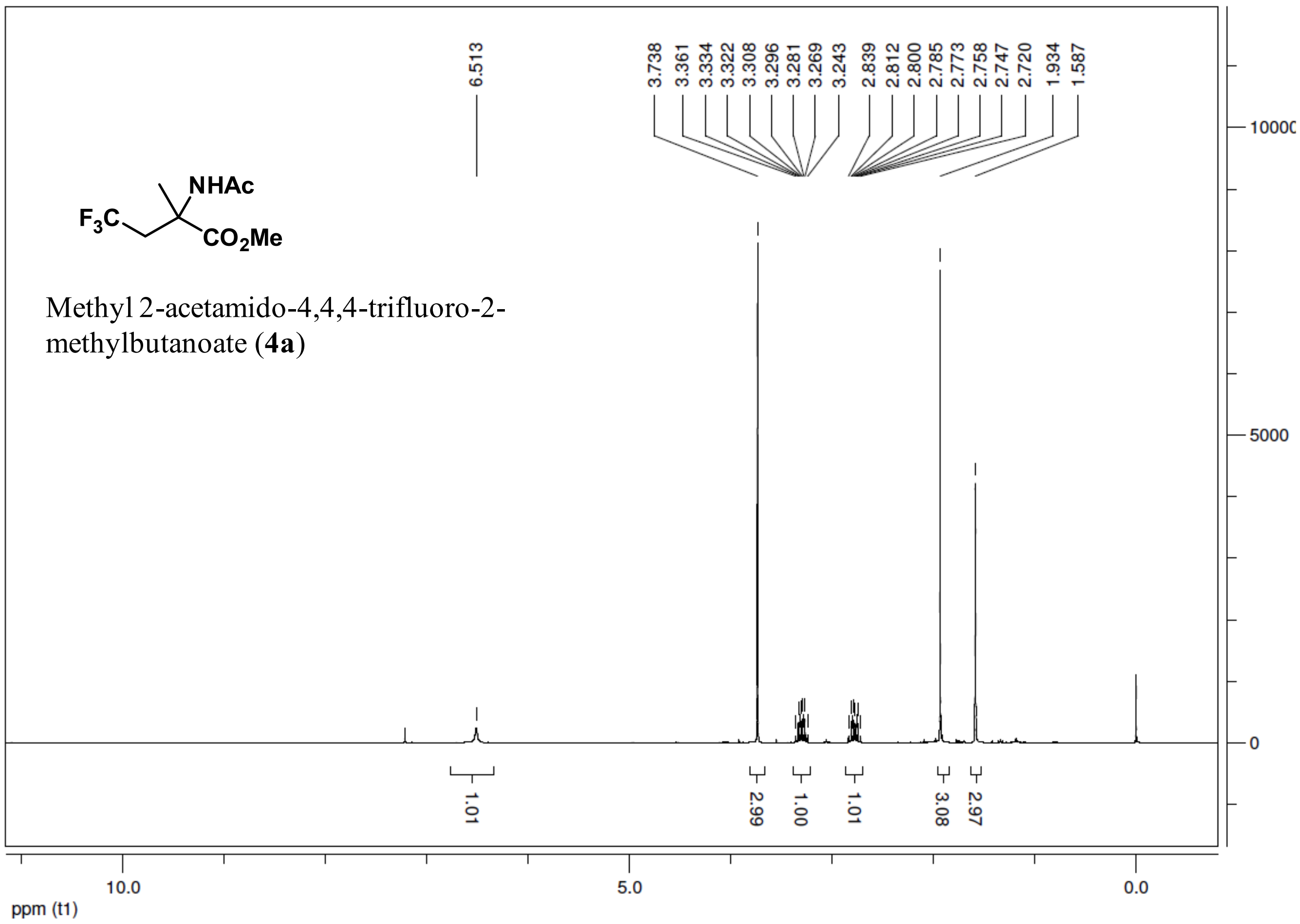
6.513
3.738
3.361
3.334
3.322
3.308
3.296
3.281
3.269
3.243
2.839
2.812
2.800
2.785
2.773
2.758
2.747
2.720
1.934
1.587
NHAc
F_3C
CO_2Me
Methyl 2-acetamido-4,4,4-trifluoro-2-methylbutanoate (**4a**)
1.01
2.99
1.00
1.01
3.08
2.97
10.0
5.0
0.0
ppm (t1)
10000
5000
0

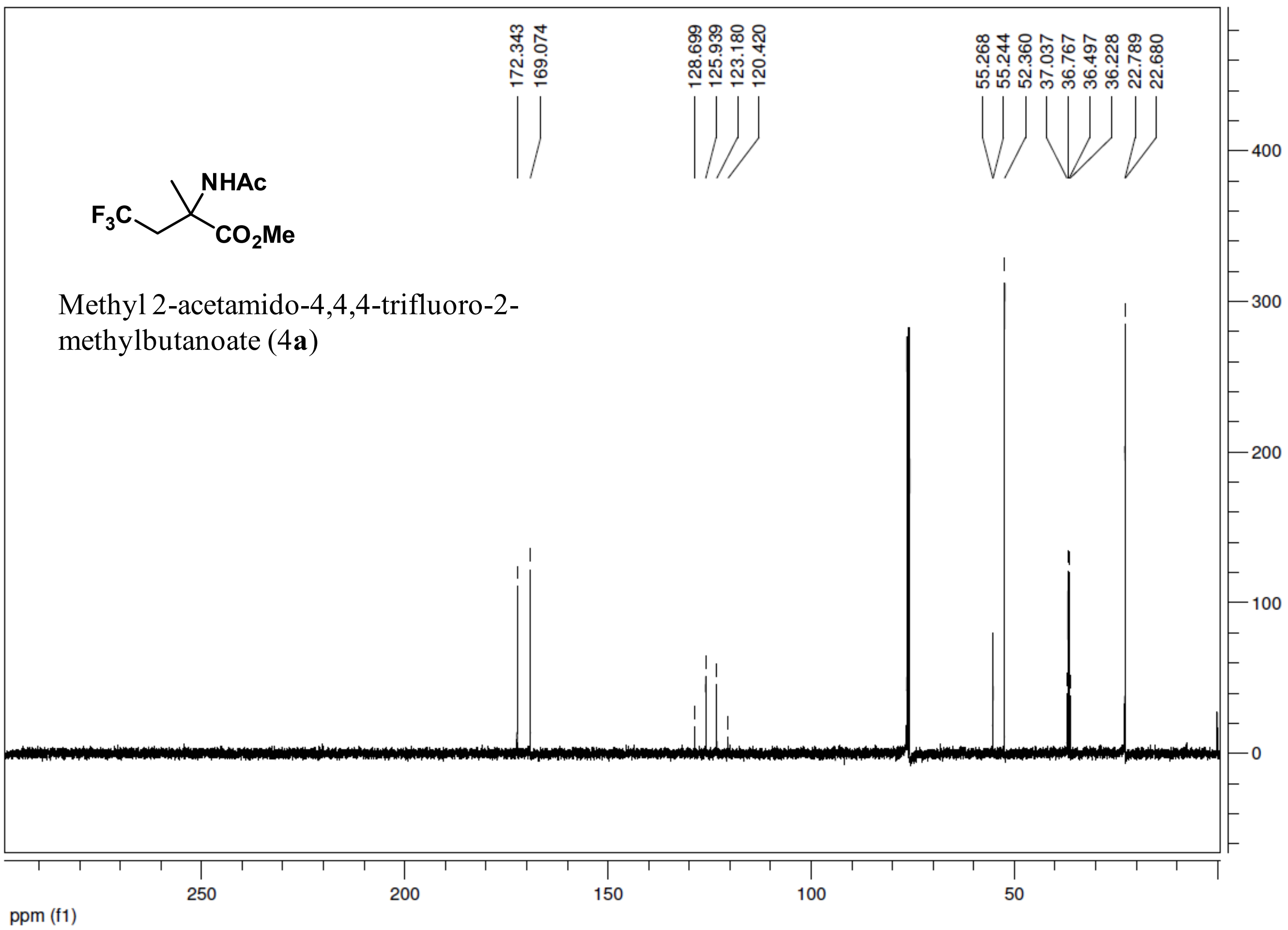
172.343
169.074
128.699
125.939
123.180
120.420
55.268
55.244
52.360
37.037
36.767
36.497
36.228
22.789
22.680
NHAc
F_3C
CO_2Me
Methyl 2-acetamido-4,4,4-trifluoro-2-methylbutanoate (4**a**)
250
200
150
100
50
ppm (f1)
400
300
200
100
0

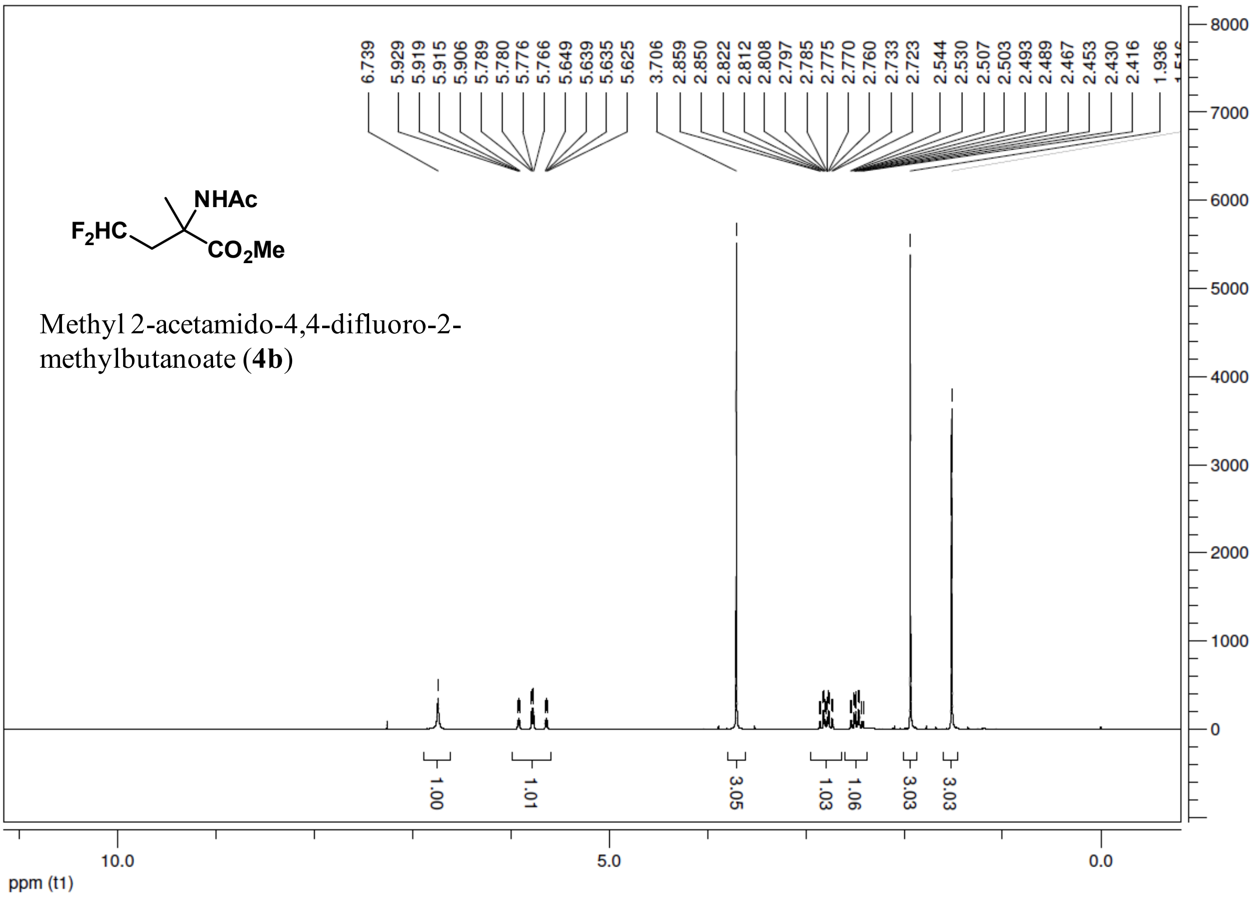
NHAc
F2HC
CO2Me
Methyl 2-acetamido-4,4-difluoro-2-methylbutanoate (4b)
6.739 5.929 5.919 5.915 5.906 5.789 5.780 5.776 5.766 5.649 5.639 5.635 5.625 3.706 2.859 2.850 2.822 2.812 2.808 2.797 2.785 2.775 2.770 2.760 2.733 2.723 2.544 2.530 2.507 2.503 2.493 2.489 2.467 2.453 2.430 2.416 1.936
1.00 1.01 3.05 1.03 1.06 3.03 3.03
10.0 5.0 0.0
ppm (t1)

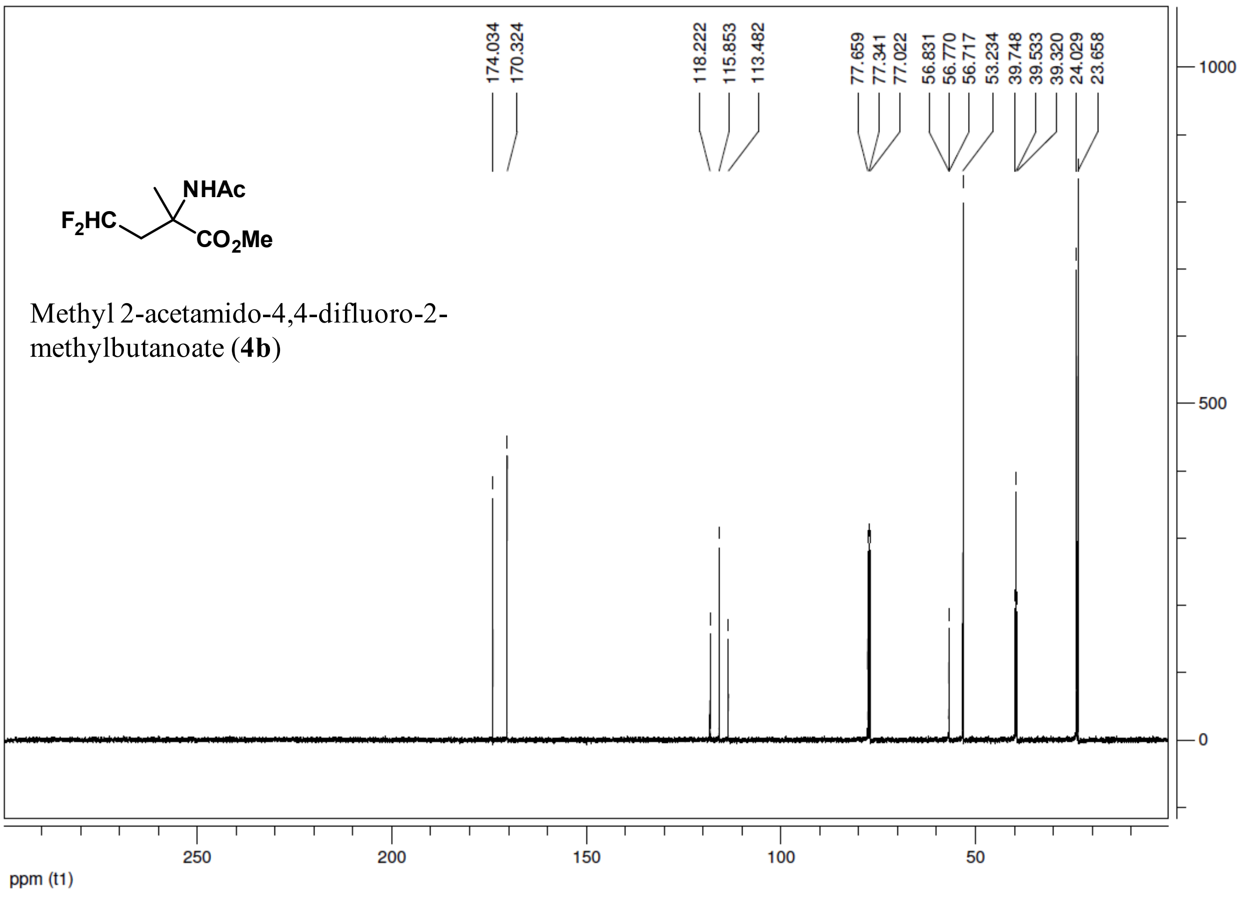
NHAc
F2HC
CO2Me
Methyl 2-acetamido-4,4-difluoro-2-methylbutanoate (4b)
174.034 170.324 118.222 115.853 113.482 77.659 77.341 77.022 56.831 56.770 56.717 53.234 39.748 39.533 39.320 24.029 23.658
250 200 150 100 50
ppm (t1)

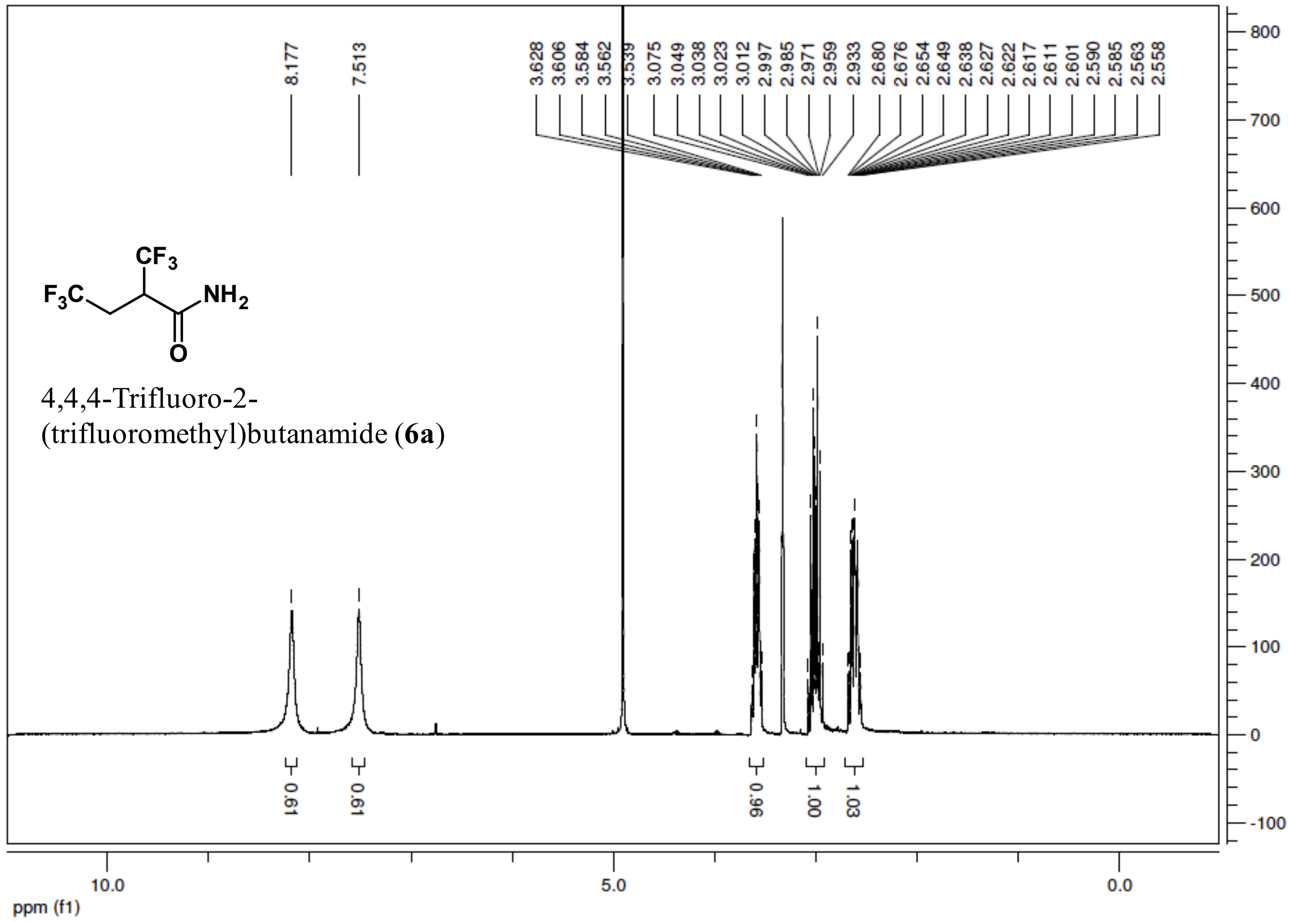
4,4,4-Trifluoro-2-(trifluoromethyl)butanamide (6a)
8.177
7.513
3.628 3.606 3.584 3.562 3.539 3.075 3.049 3.038 3.023 3.012 2.997 2.985 2.971 2.959 2.933 2.680 2.676 2.654 2.649 2.638 2.627 2.622 2.617 2.611 2.601 2.590 2.585 2.563 2.558
0.61 0.61 0.96 1.00 1.03
ppm (f1)

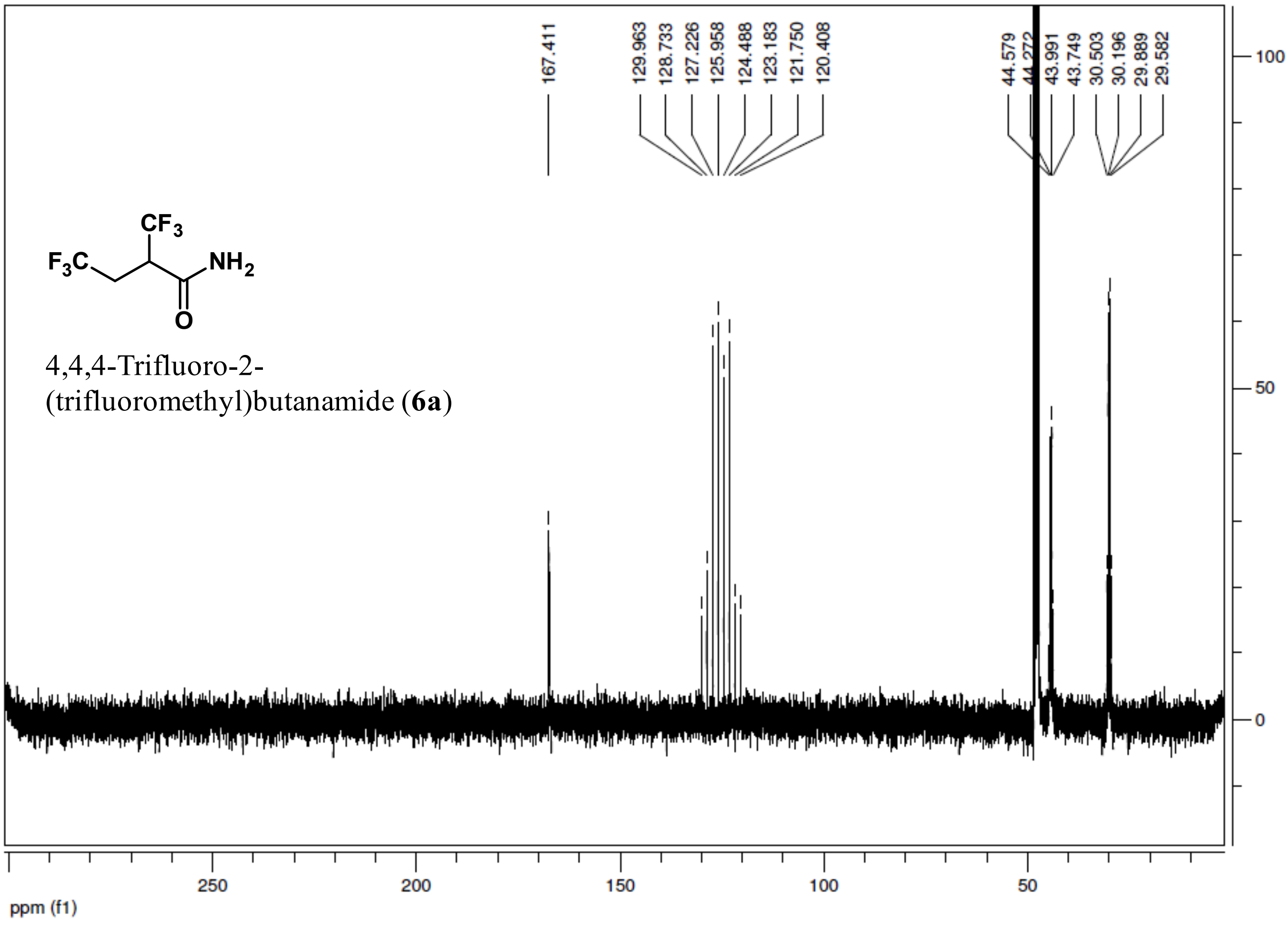
4,4,4-Trifluoro-2-(trifluoromethyl)butanamide (6a)
167.411
129.963 128.733 127.226 125.958 124.488 123.183 121.750 120.408
44.579 44.272 43.991 43.749 30.503 30.196 29.889 29.582
ppm (f1)

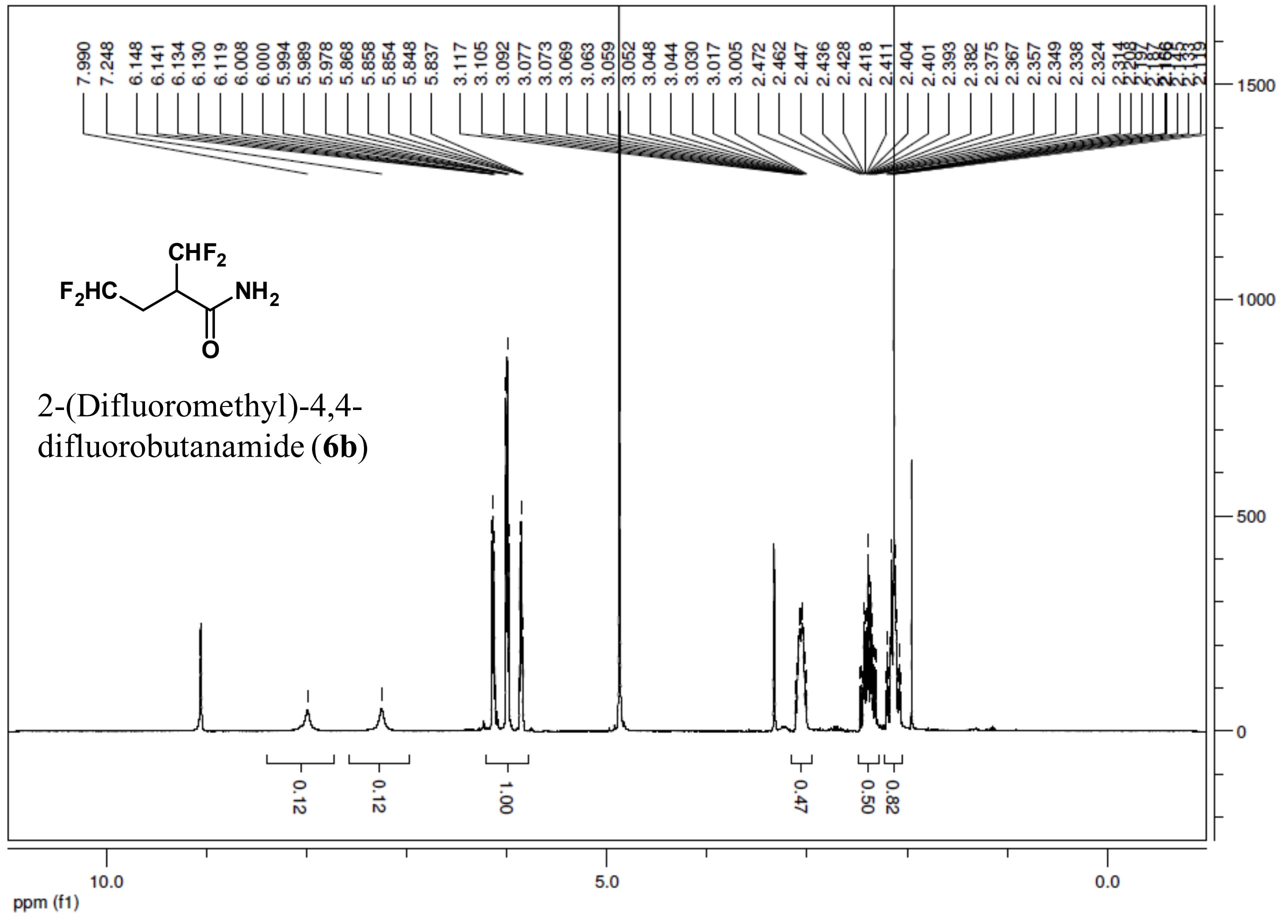
2-(Difluoromethyl)-4,4-difluorobutanamide (**6b**)
ppm (f1)

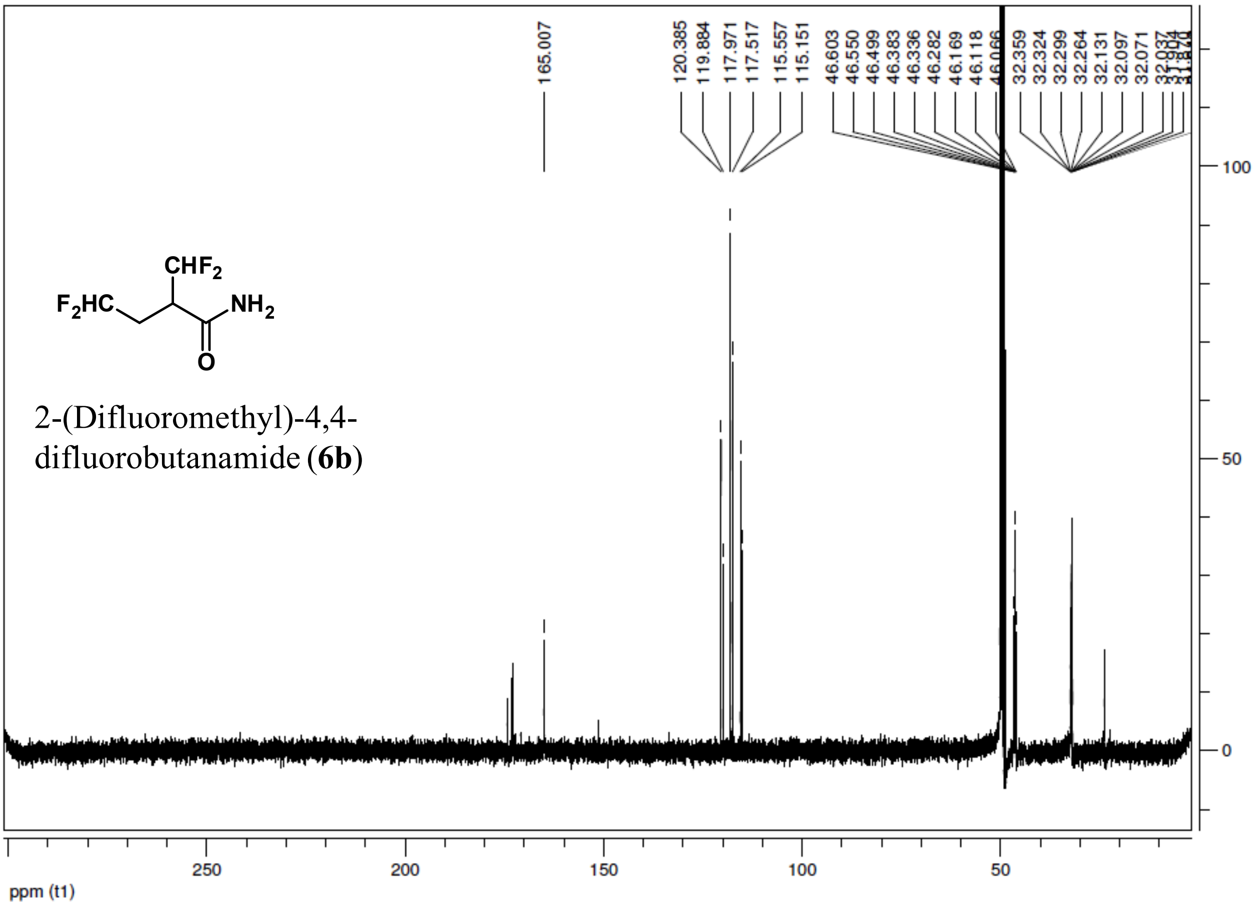
2-(Difluoromethyl)-4,4-difluorobutanamide (**6b**)
ppm (t1)

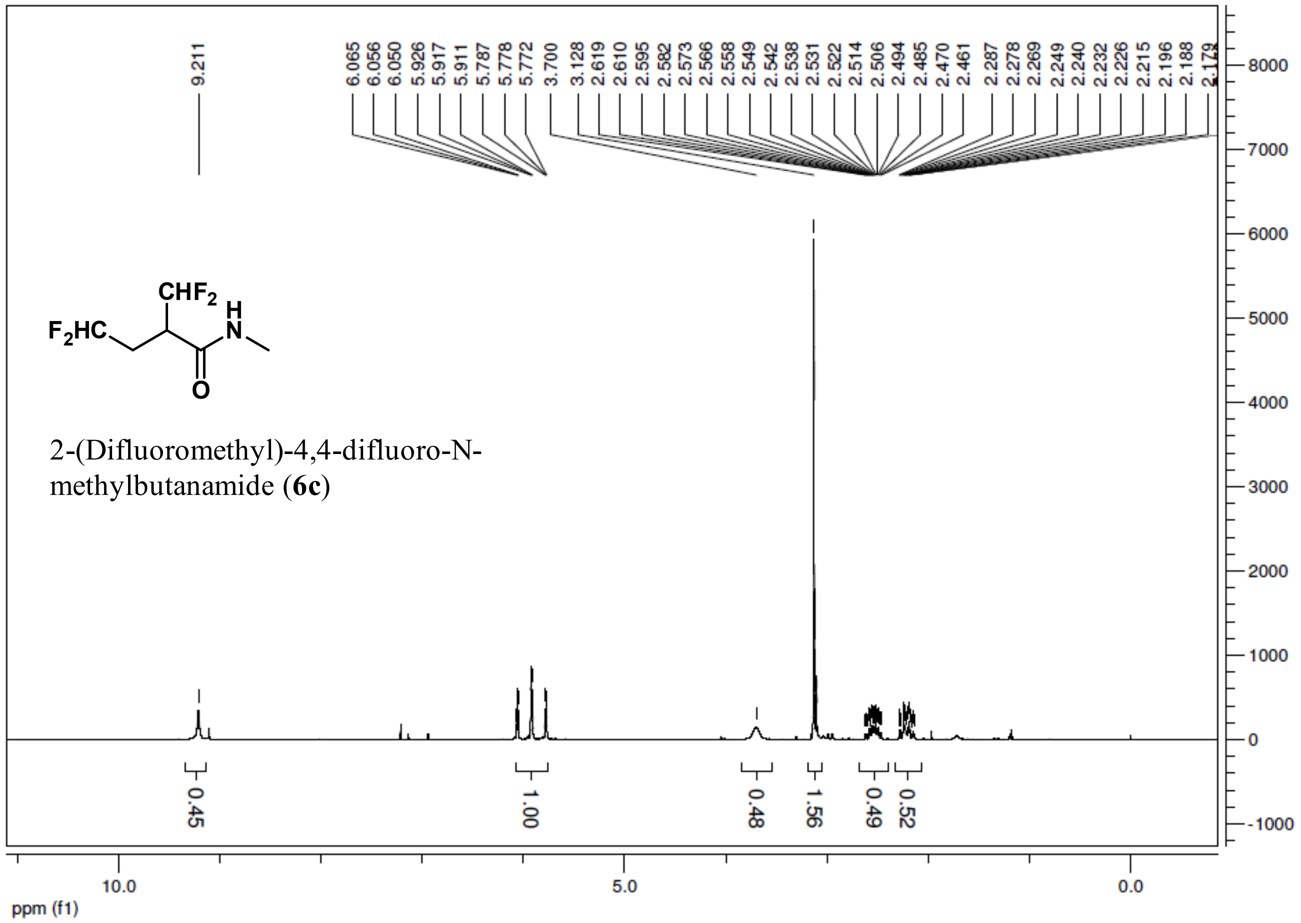
2-(Difluoromethyl)-4,4-difluoro-N-methylbutanamide (**6c**)
ppm (f1)

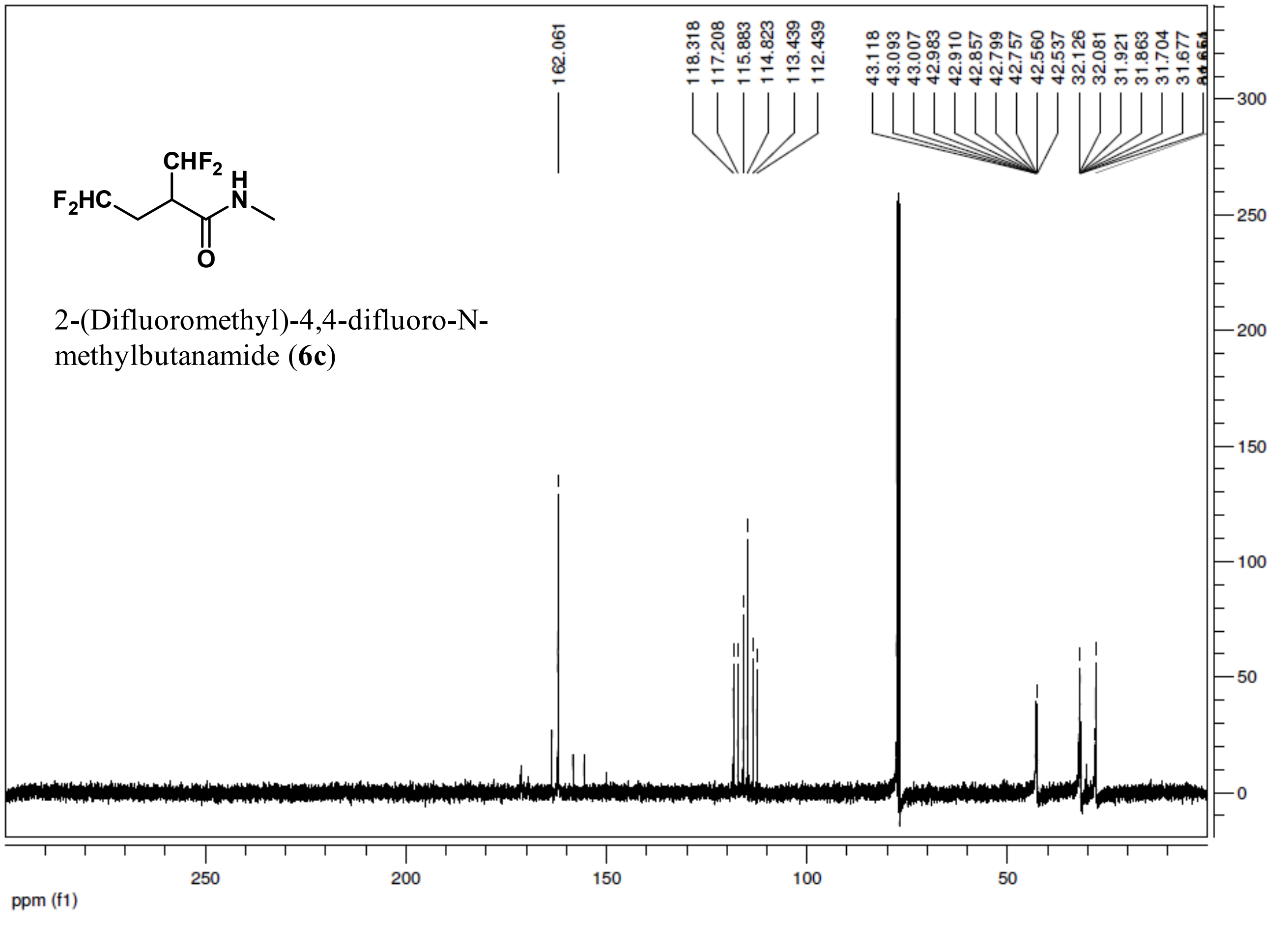
2-(Difluoromethyl)-4,4-difluoro-N-methylbutanamide (**6c**)
ppm (f1)
